# Inverse design enables large-scale high-performance meta-optics reshaping virtual reality

**DOI:** 10.1038/s41467-022-29973-3

**Published:** 2022-05-03

**Authors:** Zhaoyi Li, Raphaël Pestourie, Joon-Suh Park, Yao-Wei Huang, Steven G. Johnson, Federico Capasso

**Affiliations:** 1 Harvard John A. Paulson School of Engineering and Applied Sciences, Harvard University, Cambridge, MA USA; 2 Department of Mathematics, Massachusetts Institute of Technology, Cambridge, MA USA; 3 Nanophotonics Research Center, Korea Institute of Science and Technology, Seoul, Republic of Korea; 4 Department of Electrical and Computer Engineering, National University of Singapore, Singapore, Singapore; 5 Department of Photonics, National Yang Ming Chiao Tung University, Hsinchu, Taiwan

**Keywords:** Sub-wavelength optics, Metamaterials

## Abstract

Meta-optics has achieved major breakthroughs in the past decade; however, conventional forward design faces challenges as functionality complexity and device size scale up. Inverse design aims at optimizing meta-optics design but has been currently limited by expensive brute-force numerical solvers to small devices, which are also difficult to realize experimentally. Here, we present a general inverse-design framework for aperiodic large-scale (20k × 20k *λ*^2^) complex meta-optics in three dimensions, which alleviates computational cost for both simulation and optimization via a fast approximate solver and an adjoint method, respectively. Our framework naturally accounts for fabrication constraints via a surrogate model. In experiments, we demonstrate aberration-corrected metalenses working in the visible with high numerical aperture, poly-chromatic focusing, and large diameter up to the centimeter scale. Such large-scale meta-optics opens a new paradigm for applications, and we demonstrate its potential for future virtual-reality platforms by using a meta-eyepiece and a laser back-illuminated micro-Liquid Crystal Display.

## Abstract (web summary)

The authors present a general inverse-design framework for large-area 3D meta-optics that show engineered focusing. Such meta-optics, in combination with a laser-illuminated micro-LCD, open a path towards a future virtual reality platform.

## Introduction

Meta-optics, a new class of planar optics, has reshaped the engineering of electromagnetic waves by using artificial subwavelength components or “meta-atoms”^1–6^. Recent breakthroughs in the physics^7–11^ and advancements in large-scale meta-optics fabrication^12–14^ inspire a vision for a future in which meta-optics will be widely used. Recent studies have demonstrated cutting-edge technologies based on meta-optics platforms, such as polarization/light-field/depth imaging cameras^15–18^, metasurface-driven OLEDs^19^, virtual/augmented reality systems^20,21^, compact spectrometers^22–24^, etc. So far, the mainstream design of the meta-optics is mostly based on a “forward” methodology, in which one engineers each individual meta-atom component (as a phase shifter) independently, according to a predefined phase profile^25,26^. Forward design has demonstrated success in realizing simple device functions, like single-wavelength wave bending^27–29^ or focusing;^30,31^ however, it heavily relies on a priori intuitive knowledge and limits the development of large-scale complex meta-optics that can realize multiple custom functions depending on wavelengths, polarizations, spins, and angles of incident light. As the complexity, diameter, or constraints of a design problem scale up, the ability of a forward-driven method to search for an optimal solution becomes weaker and weaker. The future advancement of meta-optics demands a breakthrough in design philosophy.

In contrast to forward design, inverse design starts with desired functions and optimizes design geometries using computational algorithms. It has been a useful tool in solving large-scale complex engineering problems such as optimizing the shape of bridges or aircraft wings. In recent years, inverse design has been reshaping the landscape of photonics engineering. Multiple flavors of inverse design techniques have been studied: topological optimization techniques, which use a local gradient-based optimization tool to search for optimal photonic geometries^32,33^; and, machine-learning techniques^34–36^, which train a neural network to find a design for a given response^37^ or train a generative network (e.g., generative adversarial network) to sample the high-performance designs^38^. A recent evolution of inverse design in photonics optimizes the geometry and the post-processing parameters end-to-end^39–41^. Inverse design has demonstrated significant success in optimizing photonic crystals^42^, on-chip nanophotonics^43,44^, metasurfaces^45,46^, and other devices.

Inverse design remains very challenging for aperiodic large-scale meta-optics. The optimization relies on many iterations of simulations, which become computationally intractable as design dimension scales up due to the multiscale nature of design problems^47^: the nanoscale meta-atom (nm) and the macroscale meta-optics (100 s of µm to cm). On the one hand, it is unrealistic to model an aperiodic 3D device with a 1-cm diameter using the finite-difference time-domain (FDTD) or the finite element analysis method, which can capture physics at nanoscale but are limited by both computation time and memory capacity. For example, it takes ~100 h in time and ~100 gigabytes in RAM memory for a FDTD solver to simulate a metasurface device of 50 µm^2^ in size (assuming a 5-nm mesh size). On the other hand, ray-tracing simulations, which are suitable for large-scale optics design, cannot capture the full wave nature of the optical field. They also only allow slowly varying phase profiles, excluding the rich physics of rapidly varying phase wavefronts offered by engineered meta-atoms. To our knowledge, the diameter of inverse-designed fully three-dimensional metasurfaces has been limited to about 200*λ*^48–51^, about 100 µm for visible light. In addition, our inverse-design framework handles fabrication constraints inside a surrogate model, in contrast with most inverse-design frameworks, which need to add these constraints during optimization^52^.

In this paper, we present a generic inverse-design framework that enables aperiodic large-scale three-dimensional complex-function meta-optics compatible with fabrication constraints. Our inverse-design method is computationally tractable (requiring only a few hours using a desktop single-core CPU) and advantageous for macroscale (>1000 s of *λ*s) meta-optics design in tandem with exploitation of physics at the nanoscale. It greatly expands optical design to an unprecedented regime where conventional forward design is of limited use. The present design framework handles three-dimensional simulations with six orders of magnitude more parameters than the proof-of-concept two-dimensional work^53^. It controls the full polarization in contrast with ref. ^21^, which is fundamentally limited to polarization-converted light from left-handed circularly polarized (LCP) state to right-handed circularly polarized (RCP) state. These unique inverse design features enable experimental demonstration of meta-optics with high numerical aperture (NA = 0.7) and complex functionality. For example, we show polarization-insensitive RGB-achromatic metalenses and even polychromatic metalenses. These inverse-designed meta-optics realizes mm to cm scale aperture size, which corresponds to an increase of four orders of magnitude in area compared with the state of the art. To prove the potential of large-scale meta-optics in applications, we further demonstrate a meta-optics-based virtual-reality (VR) platform.

## Results

### Inverse design theoretical framework

Fundamentally different from conventional forward design, the philosophy of inverse design is to start with the goal and then optimize it given the application’s constraints. For the design of lenses, the goal is to maximize the intensity at the focal spot; that is, we maximize $\,I({\vec{{{{{{\bf{x}}}}}}}}_{{{{{{\bf{target}}}}}}}{{{{{\boldsymbol{,}}}}}}\,\vec{{{{{{\bf{p}}}}}}})$ over a vector $\vec{{{{{{\bf{p}}}}}}}$ of geometric parameters defining the metasurface, where ${\vec{{{{{{\bf{x}}}}}}}}_{{{{{{\bf{target}}}}}}}$ is the location of the focal spot^53^. For polychromatic lens design, the objective function finds a satisfying locally optimal geometry that maximizes the minimum intensity across the design wavelengths: ${\max }(\mathop{{{\min }}}\nolimits_{\lambda \in {\lambda }_{s}}({I}_{\lambda }({\vec{{{{{{\bf{x}}}}}}}}_{{{{{{\bf{target}}}}}}},\vec{{{{{{\bf{p}}}}}}})))$, where *λ*_*s*_ is a discrete set of wavelengths of interest and *I*_*λ*_ is the intensity function for a wavelength *λ*^53^. This maximizes the focal intensity at multiple wavelengths simultaneously. We further reformulate this function to be differentiable as shown in the SI.

**Fig. 1:**
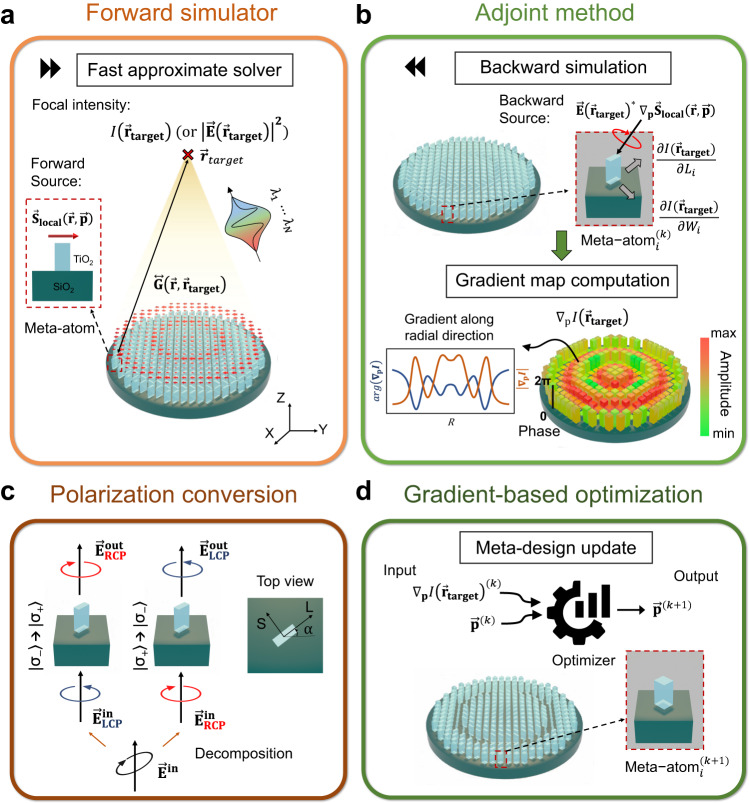
Meta-optics inverse-design framework. **a** Forward simulator by fast approximate solver, which evaluates the intensity of the field at the target via a convolution of the equivalent current with the appropriate Green’s function. **b** Adjoint method that computes the gradient with respect to all the design parameters of the metasurface at a cost of a single simulation with a customized backward source. Here, *Li* and *Wi* denote the length and width of the ith meta-atom; **c** Polarization conversion by meta-atoms as described by Jones’ matrix. **d** a gradient-based optimization method, which updates the metasurface design through iterations.

Fast and accurate “forward” evaluation of meta-optics performance is key to large-scale inverse design. We introduce a three-dimensional (3D) fast approximate solver that is based on the convolution of local fields and Green’s function (Fig. 1a). Accurate local fields above a training set of meta-atoms are computed in advance using rigorous coupled wave analysis (RCWA). A surrogate model, which is based on Chebyshev interpolation^54^, is then built to rapidly predict the local field of an arbitrary meta-atom with fabricable parameters (SI). Our surrogate model is six orders of magnitude faster than a direct simulation using RCWA (SI). It also uses Chebyshev regression (least-square smoothing) to avoid artificial oscillations (SI)^54^. By the equivalence principle, we convert the local fields to “artificial” sources of magnetic current density ${\vec{{{{{{\bf{S}}}}}}}}_{{{{{{\bf{local}}}}}}}\left(\vec{{{{{{\bf{x}}}}}}}{{{{{\boldsymbol{,}}}}}}\,\vec{{{{{{\bf{p}}}}}}}\right)$, and the focal intensity is computed by using a convolution between the current sources and vectorial Green’s function (Eq. (1))^53^:

1 $${\big|\vec{{{{{{\bf{E}}}}}}}\big({\vec{{{{{{\bf{x}}}}}}}}_{{{{{{\bf{target}}}}}}}\big)\big |}^{{{{{{\boldsymbol{2}}}}}}}={\left|{\int }_{\Sigma }{\vec{{{{{{\bf{S}}}}}}}}_{{{{{{\bf{local}}}}}}}\left(\vec{{{{{{\bf{x}}}}}}},\vec{{{{{{\bf{p}}}}}}}\right)\odot \mathop{{{{{{\bf{G}}}}}}}\limits^{\leftrightarrow}\big(\vec{{{{{{\bf{x}}}}}}},{\vec{{{{{{\bf{x}}}}}}}}_{{{{{{\bf{target}}}}}}}\big){{{{{\rm{d}}}}}}\vec{{{{{{\bf{x}}}}}}}\right|}^{{{{{{\boldsymbol{2}}}}}}}$$

where $\vec{{{{{{\bf{E}}}}}}}\big({\vec{{{{{{\bf{x}}}}}}}}_{{{{{{\bf{target}}}}}}}\big)$ is the electric field at the focal spot, **⊙** represents the Hadamard product, and $\mathop{{{{{{\bf{G}}}}}}}\limits^{\leftrightarrow}\big(\vec{{{{{{\bf{x}}}}}}}{{{{{\boldsymbol{,}}}}}}\,{\vec{{{{{{\bf{x}}}}}}}}_{{{{{{\bf{target}}}}}}}\big)$ is the dyadic Green’s function from a local position $\vec{{{{{{\bf{x}}}}}}}$ to a target position ${\vec{{{{{{\bf{x}}}}}}}}_{{{{{{\bf{target}}}}}}}$. Note that the Green’s function only needs to be computed once and can be reused in subsequent optimization iterations. It is analytical in free space and does not require a paraxial approximation. Here, we use a local periodic approximation (LPA) to predict the local fields, assuming neighboring meta-atoms are similar^53,55^. LPA is validated for our design by the agreement of predicted and experimental results; this is expected in the moderate-NA regime where the meta-atoms vary slowly over most of the surface (further discussed in the SI). To further speed up our simulator, we impose cylindrical symmetry on the design parameters while retaining the tiling of the meta-atoms in Cartesian coordinates (see SI). Unlike fully axis-symmetric designs^51^, however, on a subwavelength scale our meta-atoms break cylindrical symmetry.

**Fig. 2:**
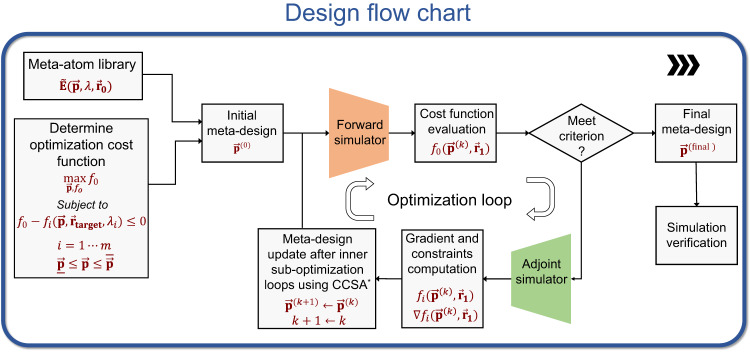
Meta-optics inverse-design flow chart. With prior knowledge of the meta-atom library and optimization problem, we start with a random metasurface design and then update the design through optimization loops that consists of a forward simulator and an adjoint-based optimization engine. Once the criterion is met, we terminate the design loop and validate the design in simulation. Note: CCSA is short for conservative convex separable approximation.

Optimization in a high-dimensional design space, when $\vec{{{{{{\bf{p}}}}}}}$ is of dimension » 1000, is another challenge for inverse design. Here, we use a local gradient-based optimization method, called a “conservative convex separable approximation”^56^, to search for an optimal design consisting of 10^6^ to 10^9^ degrees of freedom. We also applied a multi-start approach by exploring multiple random initial design parameters^57^. In our case, an initial design using the phase-matching method is not possible since the aperture of the metalens is so large (up to 20k *λ* in diameter) that the required group delay^11^ to simultaneously satisfy the phase profiles of multiple wavelengths is three orders of magnitude larger than what a single-layer meta-atom can provide. For fast computation of the gradients ${\nabla }_{{{{{{\bf{p}}}}}}}I\big({\vec{{{{{{\bf{x}}}}}}}}_{{{{{{\bf{target}}}}}}}\big)$, we take advantage of an adjoint method^58^, which can evaluate the gradients for all parameters simultaneously using only two simulations (Eq. (2)). In comparison, a traditional brute-force method needs (*N* + 1) simulations, where *N* is the dimension of $\vec{{{{{{\bf{p}}}}}}}$. The adjoint method is illustrated in Fig. 1b (details in the SI):

2 $${\nabla }_{{{{{{\bf{p}}}}}}}I\big({\vec{{{{{{\bf{x}}}}}}}}_{{{{{{\bf{target}}}}}}}\big)=2{\mathfrak{R}}\left({\int }_{\Sigma }\left({\vec{{{{{{\bf{E}}}}}}}{\big({\vec{{{{{{\bf{x}}}}}}}}_{{{{{{\bf{target}}}}}}}\big)}^{* }{\nabla }_{{{{{{\bf{p}}}}}}}\vec{{{{{{\bf{S}}}}}}}}_{{{{{{\bf{local}}}}}}}\left(\vec{{{{{{\bf{x}}}}}}},\vec{{{{{{\bf{p}}}}}}}\right)\right)\odot \mathop{{{{{{\bf{G}}}}}}}\limits^{\leftrightarrow}\big(\vec{{{{{{\bf{x}}}}}}},\,{\vec{{{{{{\bf{x}}}}}}}}_{{{{{{\bf{target}}}}}}}\big){{{{{\rm{d}}}}}}\vec{{{{{{\bf{x}}}}}}}\right)$$

where ℜ denotes the real part, $\mathop{{{{{{\bf{G}}}}}}}\limits^{\leftrightarrow}\big(\vec{{{{{{\bf{x}}}}}}}{{{{{\boldsymbol{,}}}}}}\,{\vec{{{{{{\bf{x}}}}}}}}_{{{{{{\bf{target}}}}}}}\big)$ is the dyadic Green’s function from a target position ${\vec{{{{{{\bf{x}}}}}}}}_{{{{{{\bf{target}}}}}}}$ to a local position $\vec{{{{{{\bf{x}}}}}}}$, and ${{\nabla }_{{{{{{\bf{p}}}}}}}\vec{{{{{{\bf{S}}}}}}}}_{{{{{{\bf{local}}}}}}}\left(\vec{{{{{{\bf{x}}}}}}}{{{{{\boldsymbol{,}}}}}}\vec{{{{{{\bf{p}}}}}}}\right)$ is the gradient of the local current source with respect to the design parameter $\vec{{{{{{\bf{p}}}}}}}$, which can also be fast evaluated by using a pre-trained surrogate model at low cost. It means the gradient ${\nabla }_{{{{{{\bf{p}}}}}}}I\big({\vec{{{{{{\bf{x}}}}}}}}_{{{{{{\bf{target}}}}}}}\big)$ can be efficiently obtained everywhere at once in a backward simulation using an equivalent source $\big({\vec{{{{{{\bf{E}}}}}}}{\big({\vec{{{{{{\bf{x}}}}}}}}_{{{{{{\bf{target}}}}}}}\big)}^{{{{{{\boldsymbol{* }}}}}}}{\nabla }_{{{{{{\bf{p}}}}}}}\vec{{{{{{\bf{S}}}}}}}}_{{{{{{\bf{local}}}}}}}\left(\vec{{{{{{\bf{x}}}}}}}{{{{{\boldsymbol{,}}}}}}\vec{{{{{{\bf{p}}}}}}}\right)\big)$. The gradient information was then fed into the optimizer for meta-design update (Fig. 1d). The whole design flow is summarized in Fig. 2. We started from a random meta-design and went through iterations of optimization loops, relying on a forward simulator and an adjoint simulator, until the device performance converged and met the design criteria. We then evaluated the final design in simulations and further in experiment.

### Large-scale inverse-designed polychromatic metalenses

**Fig. 3:**
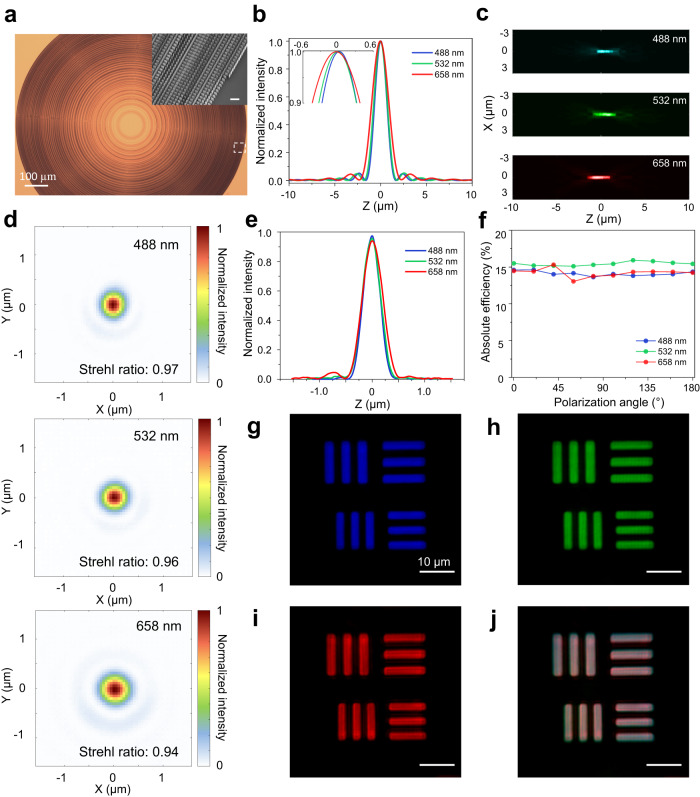
2-mm-diameter RGB-achromatic polarization-insensitive metalens (NA = 0.7). **a** Optical microscope image of the fabricated device. Scale bar is 100 µm. The inset is the SEM image that corresponds to the region within the white dashed box. Scale bar is 1 µm. **b** Simulations of the normalized focal intensity along the optical axis (*Z*) at the three design RGB wavelengths. The inset shows the zoomed-in view of the intensity peaks. **c** Measured focal intensity distribution in the *XZ* plane. **d** Measured focal plane intensity distribution at *λ* = 488, 532, and 658 nm (from top to bottom, respectively). **e** Measured focal intensity profile of RGB focal spots. The peak values are normalized according to Airy functions (SI). **f** Measured focusing efficiency as function of polarization angle of the incident light, showing polarization-insensitive focusing. **g**–**i** Imaging results of the element #5 and #6 from the group #7 of the USAF resolution target at *λ* = 488, 532, and 658 nm, respectively. The scale bar is 10 µm unless noted. **j** Imaging result under synthesized white light illumination using RGB light.

Engineering a focus at multiple wavelengths and in different polarization states simultaneously is challenging, especially in the case of high NA. By applying the inverse-design method, we first demonstrated a polarization-insensitive, RGB-achromatic metalens. This metalens has a diameter of 2 mm and numerical aperture (NA) of 0.7. Figure 3a is an optical microscope image of the device fabricated using electron beam lithography (EBL) and atomic layer deposition^59^. The inset scanning electron microscope image shows anisotropic TiO_2_ nanofin structures with spatially varying inverse-designed geometries on top of a fused-silica substrate. The height of the nanofins is 600 nm and the square-lattice periodicity is 400 nm. Each nanofin has a rectangular shape whose sizes are determined by optimization and is aligned parallel to the unit-cell axes. It partially converts LCP light to RCP light, and vice versa (Fig. 1c). The polarization conversion from L (|*σ*_−_〉) to R (|*σ*_+_〉) and R to L is equal by symmetry in our case, as described by the Jones’ matrix (Eq. (3)):

3 $$\left[\begin{array}{c}{\widetilde{{{{{{\bf{E}}}}}}}}_{{{{{{\bf{LCP}}}}}}}^{{{{{{\bf{out}}}}}}}\\ {\widetilde{{{{{{\bf{E}}}}}}}}_{{{{{{\bf{RCP}}}}}}}^{{{{{{\bf{out}}}}}}}\end{array}\right]=\left[\begin{array}{cc}\kern-1.5pc ({\widetilde{{{{{{\bf{t}}}}}}}}_{{{{{{\bf{L}}}}}}}+{\widetilde{{{{{{\bf{t}}}}}}}}_{{{{{{\bf{s}}}}}}})/2 & \kern1.6pc({\widetilde{{{{{{\bf{t}}}}}}}}_{{{{{{\bf{L}}}}}}}-{\widetilde{{{{{{\bf{t}}}}}}}}_{{{{{{\bf{s}}}}}}}){e}^{-2i\alpha }/2\\ ({\widetilde{{{{{{\bf{t}}}}}}}}_{{{{{{\bf{L}}}}}}}-{\widetilde{{{{{{\bf{t}}}}}}}}_{{{{{{\bf{s}}}}}}}){e}^{+2i\alpha }/2 & ({\widetilde{{{{{{\bf{t}}}}}}}}_{{{{{{\bf{L}}}}}}}+{\widetilde{{{{{{\bf{t}}}}}}}}_{{{{{{\bf{s}}}}}}})/2\end{array}\right]\left[\begin{array}{c}{\widetilde{{{{{{\bf{E}}}}}}}}_{{{{{{\bf{LCP}}}}}}}^{{{{{{\bf{in}}}}}}}\\ {\widetilde{{{{{{\bf{E}}}}}}}}_{{{{{{\bf{RCP}}}}}}}^{{{{{{\bf{in}}}}}}}\end{array}\right]$$

Where ${\widetilde{{{{{{\bf{t}}}}}}}}_{{{{{{\bf{L}}}}}}}$ and ${\widetilde{{{{{{\bf{t}}}}}}}}_{{{{{{\bf{s}}}}}}}$ are complex transmission along long and short axis, respectively, *α* is the rotation angle of nanofin, “out” means output field, and “in” means input field. Due to this symmetry and the fact that any polarization state can be written as superposition of LCP and RCP fields, our metalens design can focus light equally well for any arbitrary polarization state^59,60^. Figure 3b shows the simulation results for the focal intensity distribution along the optical axis at the design RGB wavelengths of 488, 532, and 658 nm. These wavelengths are chosen to correspond to our single-wavelength laser diodes. The inset is the zoomed-in view of the focal peaks, which shows achromatic focusing with negligible focal shifts (<50 nm). Figure 3c is the measured focal intensity distribution at the RGB wavelengths in the *XZ* plane, where *X* is along the lens radial direction and *Z* is along the optical axis. The maximum focal shift is ~1.5 µm, which is ~0.15% of the focal length. Figure 3d, from top to bottom, is the measured intensity distribution at the focal planes of the blue, green, and red wavelengths, respectively. Their respective measured focal intensity profiles (Fig. 3e) imply diffraction-limited focus (detailed analysis can be found in the SI). We measured the absolute focusing efficiency, which is defined as the ratio between the power in the focal spots and the incident power, as a function of the incidence polarization angles. Figure 3f shows that the absolute efficiency is about 15% at RGB wavelengths and is independent of the polarization angle of the incident light. Moreover, our metalens focuses light of an arbitrary polarization state to its orthogonal state, which is useful for improving the imaging contrast. We further characterized the imaging performance of the metalens using the United States Air Force (USAF) resolution target. Figure 3g–i is the imaging result of the element No. 5 and No. 6 from group No. 7 under blue, green, and red illumination. The smallest feature size is 2.2 µm and can be clearly resolved. To demonstrate achromatic imaging, we further imaged the same area using synthesized white-light illumination by mixing RGB color in the incident light. The result is a clear whitish image with the same magnification (Fig. 3j). More imaging results under other synthesized light illumination can be found in SI.

The inverse-design method has more pronounced advantages over conventional forward design methods when designing a meta-optics with more complicated functions. Forward design methods, like wavefront phase-matching, struggle in the regime where no single meta-atom can simultaneously satisfy the targeted phase profiles for multiple functions. They cannot balance compromises between meta-atoms systematically because they optimize each meta-atom separately. A good phase-matching will try to reduce the overall phase errors, at the risk of omitting one of the functions as well as ignoring cross-talks between functions. They also neglect the effect of a non-uniform amplitude or phase profile. A good phase-matching sometimes comes at the cost of low efficiency due to the intrinsic correlation between the phase and amplitude of the engineered electromagnetic wave by meta-atoms. Moreover, forward designs are usually one-way without feedback loops, and thus do not provide confirmation of optimality or robustness. Importantly, forward designs require a priori knowledge of the desired wave solution, which is unavailable for complex problems. In comparison, our inverse-design method can obtain previously unknown solutions to complex design problems because it starts only with the design objective and iteratively searches for an optimal solution in a hyperdimensional design space. It also evaluates the objective functions directly against design parameters and balances the non-uniform amplitude/phase profiles automatically across the metasurface to optimize complex objective functions and cross-talks.

**Fig. 4:**
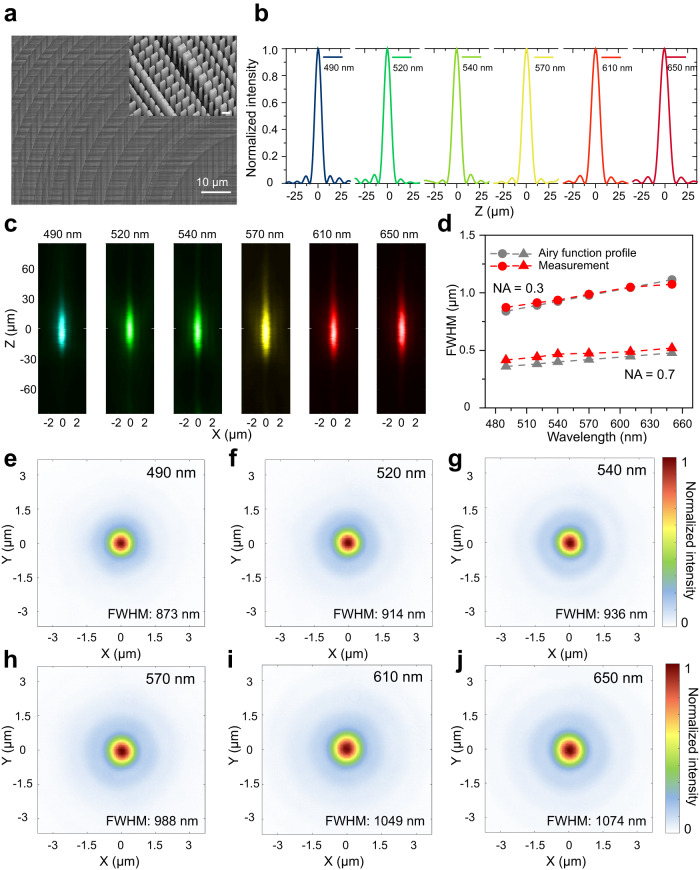
2-mm-diameter polychromatic polarization-insensitive metalenses (NA = 0.3 and 0.7). **a** SEM image of the fabricated metalens with NA = 0.3. The scale bar is 10 µm. The inset is a zoomed-in tilted view. The scale bar is 400 nm. **b** Simulations of normalized focal intensity distribution along the optical axis at six design wavelengths (NA = 0.3). **c** Measured focal intensity distribution in the *XZ* plane at six wavelengths (NA = 0.3). **d** Measured full-width-half-maximums (FWHMs) of the focal spots at six design wavelengths in comparison with ideal Airy function profile (NA = 0.3 and 0.7). **e**–**j** Measured focal plane intensity distribution at the design wavelengths (NA = 0.3).

To prove the concept, we further demonstrated an experimental polychromatic metalenses with six-wavelength-achromatic-focusing performance for visible light. These two metalenses have an aperture diameter of 2 mm and NAs of 0.3 and 0.7. Figure 4a is the SEM image of the NA = 0.3 metalens. This metalens is designed for achromatic focusing at six wavelengths of 490, 520, 540, 570, 610, and 650 nm. Figure 4b is the simulation result showing their focal intensity distribution along the optical axis. The measured focusing intensity (Fig. 4c) in the *XZ* plane shows good agreement with the simulation results. The maximum focal shift among design wavelengths is 500 nm (<0.02% of the focal length). The measured focusing efficiency is ~8%, and imaging results are shown in the SI. The simulation and measurement results of the NA = 0.7 metalens are also shown in the SI. Figure 4d shows that the measured full-width-half-maximums of the focal spots in comparison with the ideal Airy-function theory. The subtle differences are because we used a super-continuum laser as the light source, which has a larger linewidth (FMHWs) of ~5 nm in comparison with ~0.5 nm linewidth of laser diodes (SI). Figure 4e–j is the measured focal intensity distribution at a common focal plane of six design wavelengths. The measurement results of the NA = 0.7 metalens are shown in the SI.

**Fig. 5:**
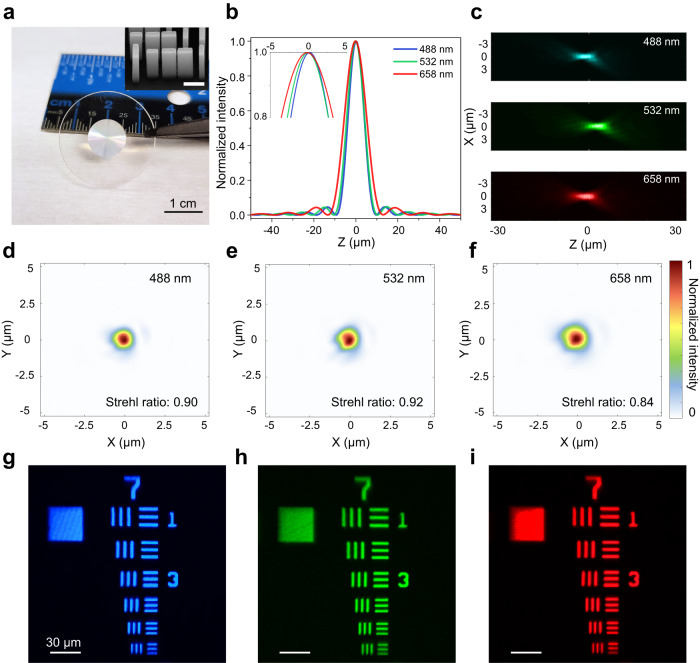
1-cm-diameter RGB-achromatic polarization-insensitive metalens (NA = 0.3). **a** Photograph of the fabricated 1-cm-diameter metalens. The scale bar is 1 cm. The inset is the SEM image of the nanostructures used in the metalens. The scale bar is 500 nm. **b** Simulations of focal intensity distribution along the optical axis at RGB design wavelengths. The inset is the zoomed-in view of the peaks. **c** Measured focal intensity distribution in the *XZ* plane at RGB wavelengths. **d**–**f** Measured normalized focal intensity distribution at the focal planes of *λ* = 488, 532, and 658 nm, respectively. **g**–**i** Imaging results of the group #7 of the USAF resolution target under blue, green, and red-light illumination. The scale bars are 30 µm.

To further prove the scalability of our inverse-design method, we designed and fabricated a cm-scale metalens. This metalens is designed for achromatic focusing at RGB wavelengths with an NA of 0.3. Figure 5a shows the 1-cm-diameter RGB-achromatic flat meta-optics on 2-inch fused silica wafer with a reference ruler behind. The inset is the SEM image showing the meta-atoms building blocks. We utilized a fast E-beam writer and operated at a high current. Consequently, we achieved 10-nm structural resolution at a low cost in fabrication time. Figure 5b is the simulation result showing the focal intensity distribution along the optical axis at design wavelengths, and the inset is the zoomed-in view of the peaks to show its achromatic focusing performance. The measured focal intensity distribution in the *XZ* plane is shown in Fig. 5c. The maximum focal shift among RGB wavelengths is ~4.5 µm, which is ~0.03% of the design focal length. Figure 5d–f shows the measured focal intensity distribution at the focal planes of *λ* = 488 nm, 532 nm, and 658 nm. The measured focusing efficiency at RGB is around 15%, and the design simulations show ~24% (SI). The difference can be attributed to fabrication errors. For example, the stitching errors between writing fields result in reduced focusing efficiency. We discuss this result and strategies for further improvement in the final section. The slight distortion of the focal spots is due to the non-uniform incident illumination over the cm-scale lens’ aperture, which was not anticipated during design. We further characterized the metalens by imaging the whole group No. 7 of the USAF resolution targets. Figure 5g–i is the imaging result under illumination of blue, green, and red incident light, respectively, which shows excellent imaging performance.

Furthermore, we compare forward designs with our inverse design using the 1-cm-diameter RGB-achromatic polarization-insensitive metalens as a benchmark, and the simulation results are summarized in SI. The forward design results vary with the definitions of objective functions that quantify the phase-matching conditions. The corresponding focusing efficiencies are not only lower but also non-uniform at RGB wavelengths. It reveals the limitations of forward design when applied to a design problem that involves multiple objectives and is subject to multiple constraints. In comparison, the inverse design results show better (~24%) and uniform focusing efficiencies (SI). Furthermore, the inverse design can be used to mitigate ghost focal spots or reduce halo. For example, its objective function can define the light intensity distribution along the optical axis or the scattering of zeroth-order light.

### Virtual-reality imaging demonstration

Large-scale meta-optics may have significant impact on many applications. Here, we demonstrate a VR imaging system based on our meta-optics. VR is a technology that creates an immersive experience by replacing reality with an imaginary world^61^. Its recent breakthroughs have not only attracted attention from the scientific community and industry but have also piqued the interest of the general public. Unfortunately, widespread use of VR devices has been hindered by a bottleneck in the optical architecture. The eyepieces used in current VR headsets mostly rely on refractive singlets, which suffer from bulky size and weight, and they furthermore compromise the viewing experience due to spherical and chromatic aberrations^62^. Meta-optics offer a technology to address these challenges of current VR systems^21^.

**Fig. 6:**
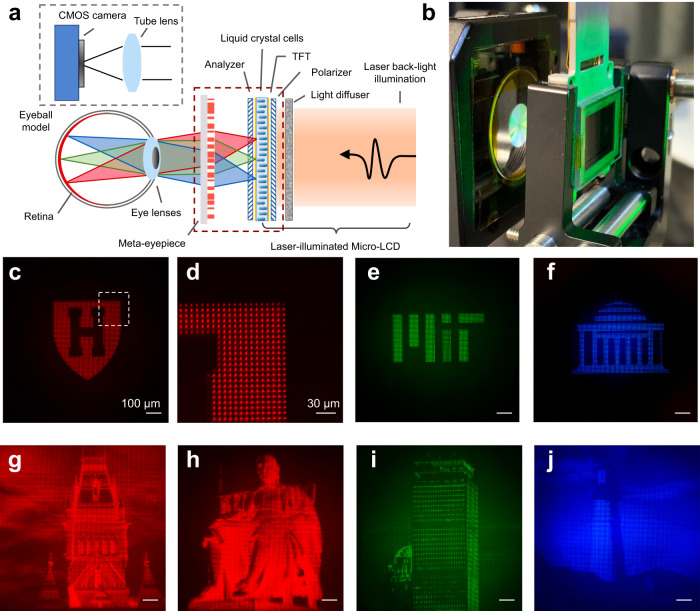
Virtual-reality imaging setup and demo using the meta-optics eyepiece. **a** Schematic of the virtual-reality near-eye projection setup comprising an RGB-achromatic meta-optic eyepiece and a laser-illuminated micro-LCD. **b** Photography of the optical setup corresponding to the red dashed line in **a**. The micro-LCD is mounted on a motorized stage and in front of the flat meta-optics. **c** Binary VR imaging result showing a Harvard logo in red color. The scale bar is 100 µm unless noted. **d** The zoomed-in view of the dash-lined area in **c**. It shows the meta-optics can resolves every single pixel of the micro-LCD. The scale bar is 30 µm. **e**, **f** Binary imaging result of MIT logos in green and blue color, respectively. **g**, **h** Grayscale VR imaging results showing a building and statue, in the Harvard campus, in red color. **i**, **j** Grayscale VR imaging results of a Boston building and a light house in green and blue color, respectively.

**Fig. 7:**
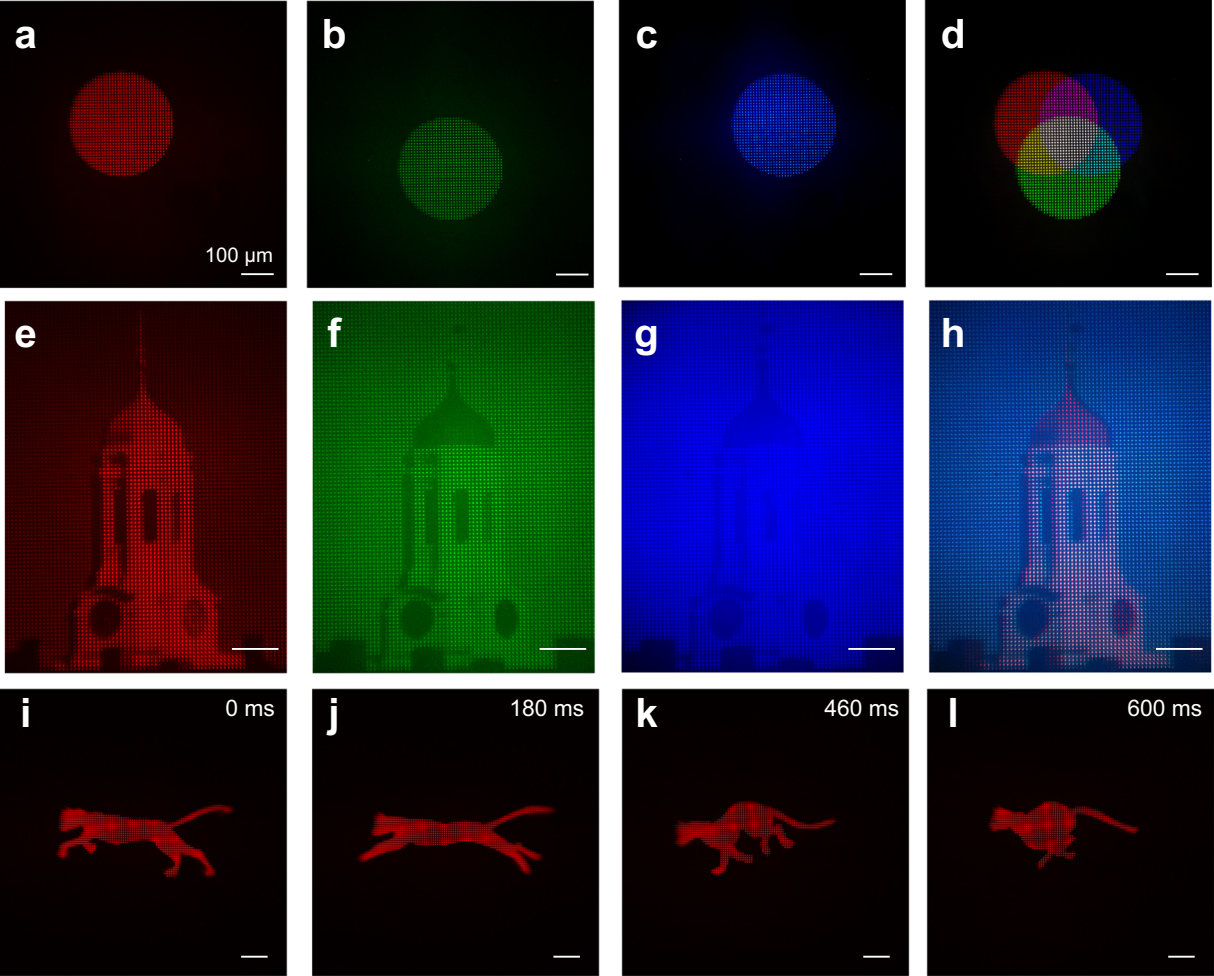
Virtual-reality imaging demo of color mixing and movie. **a**–**c** VR images of a red, green, and blue circles, respectively. The scale bar is 100 µm unless noted. **d** Simulated VR image by superposing **a**–**c**. **e**–**g** Grayscale VR imaging results of a Harvard tower in red, green, and blue channels, respectively. **h** Simulated full-color VR imaging result by combining RGB image channels shown in **e**–**g**. **i**–**l** A VR imaging movie at different frames showing a running cat. The movie can be found in the supplementary material. The near-eye display has a refresh rate of 60 Hz.

Figure 6a is the schematic of our VR system, based on our cm-scale RGB-achromatic meta-eyepiece and a laser-illuminated micro-LCD. The micro-LCD is placed close to the focal plane of the meta-eyepiece, and the image on the display is projected via the meta-eyepiece onto the retina, creating a virtual scene. In the experiment, we used a tube lens to mimic the cornea and eye crystalline lens and a CMOS camera to mimic the retina. In addition, we home-built a near-eye display using the laser light as the back-illumination source. Such a display offers high brightness and a wide color gamut due to the narrow linewidth. The pixel size is about 8 µm, matching the state of the art. Figure 6b shows the key components of the meta-eyepiece and the display as illustrated in the dashed brown box of Fig. 6a. We first demonstrate binary VR imaging. Figure 6c shows the VR image of a red letter-H shield logo, and Fig. 6d is the zoomed-in view of one corner (from the white dashed box of Fig. 6c). One can see that the meta-eyepiece resolves every pixel of the display. Figure 6e, f is the imaging result for an MIT logo under green and blue illumination, respectively. We further demonstrated grayscale VR imaging. Figure 6g, h is a grayscale imaging result (in red light) showing a Harvard building and statue, respectively. Figure 6i, j shows the grayscale VR images of a building and lighthouse in green and blue, respectively. These RGB-color imaging results imply an ability to image in full-color, because color images are simply formed by mixing these primary colors. For example, Fig. 7a–c shows VR imaging of distinct red, green, and blue circles, respectively. Figure 7d is the simulated color VR imaging result by superimposing Fig. 7a–c, which show synthesized colors of yellow, magenta, cyan, and white in the circle overlapping regions. Furthermore, Fig. 7e–g shows VR imaging of a Harvard tower in red, green, and blue channel. Figure 7h is the simulated full-color imaging result by superimposing the RGB images (Fig. 7e–g). In addition to the static VR images, our VR system can also display a dynamic VR object. Figure 7i–l displays a running cat that is captured at 0, 180, 460, and 600 ms, respectively. The near-eye display has a refresh rate of 60 Hz, and the recorded movie can be found in the SI. We further discuss a strategy to reduce form factor of the display by using a metasurface-based illumination plate (SI).

This work shows major advances over the previous VR system^21^. Thanks to the innovative inverse-design method, the meta-optics has increased the aperture size from mm to cm, which means it can be integrated with micro displays and is more realistic for applications. Micro displays are the future trend for VR optical engines; however, there has not yet been an eyepiece solution that can resolve high-resolution (~5 µm) color images. Second, the meta-optics now has polarization-insensitive focusing performance, which alleviates additional polarization-selection components (e.g., linear polarizer and phase retarder) and makes better use of incident light (focusing efficiency increases by more than double compared to ref. ^21^). Third, the meta-atoms now have a simple geometry shape and, thus, are more compatible with large-scale and mass production. Finally, displaying a movie is now possible thanks to the high refresh rate of our system. In the future, we believe meta-optics will augment conventional lens platforms^63^ to form a high-performance aberration-free compact hybrid eyepiece for VR/AR. The meta-eyepiece in this work corrects both chromatic and monochromatic aberrations under normal incidence. Future work will improve upon this to optimize corrections for higher-order aberrations such as coma and field of curvature. Possible research directions include a meta-system consisting of multiple metasurfaces pieces^47^ or a hybrid design^63^ that combines a refractive element with meta-optics.

## Discussion

In this paper, we presented a general inverse-design framework that is suitable for the large-scale 3D photonic-device optimization. We demonstrated inverse-designed 3D meta-optics of large diameters, including 2-mm-diameter RGB-achromatic and polychromatic metalenses, and even a cm-scale RGB-achromatic metalens, the largest to date, which consists of ~10^9^ meta-atoms. Furthermore, we demonstrate a path toward a future VR platform based on a meta-eyepiece and a laser-illuminated micro-LCD. This inverse-design method is also applicable to optimizing other optical elements in a VR/AR system, such as optical combiners.

In general, the development of next-generation wearable imaging platforms that have small form factor, high focusing efficiency, and correct multiple aberrations remains a challenging research topic. A recent developed “pancake lenses” for VR headsets^64^ comprising a concave half-mirror and a reflection polarizer is more compact compared to a conventional refractive eyepiece; however, the transmission efficiency is limited to ~12.5%. Our demonstrated meta-optics so far has a focusing efficiency of ~15% at RGB wavelengths under unpolarized illumination. In comparison, our previously reported 2-mm-diameter RGB-achromatic polarization-sensitive metalens (NA = 0.7) showed ~12% focusing efficiency at RGB wavelengths under LCP illumination, which is equivalent to ~6% under unpolarized illumination. Polarization-insensitive focusing of our metalens is achieved by using anisotropic meta-atoms. It means that the imaging contrast can be improved by selecting output light polarization despite relatively low focusing efficiency. In comparison, isotropic meta-optics is not ideal for multiwavelength engineering and direct imaging applications since it suffers from background light when focusing efficiency is low^13^. To reduce the power consumption of a future VR device, the focusing efficiency of our meta-eyepieces needs to be increased. Further device performance improvements require innovations in the meta-atoms, i.e., in the building blocks of the metasurface. We envision the next-generation of freeform and multiple-layered meta-atoms, which embraces more degrees of freedom and richer physics, as the key to greater performance and functionality. Implementing complex meta-atoms in a large-area inverse-design framework also requires advances in computational methods. The Chebyshev surrogate model used in this work needs an exponentially increasing dataset for more design parameters, but recent work has shown that neural networks utilizing new active-learning techniques^65^ and incorporating physics knowledge^66^ can handle ten parameters with orders of magnitude less training data. These advances mean that future surrogate-based fast solvers can use more accurate methods based on supercell domains^66^ that better capture rapid surface variations. Fully freeform topology optimization has also begun to steadily approach larger scales by exploiting domain-decomposition approximations^48,67^ and axisymmetric restrictions^51^ with the help of large-scale computing power and fast surrogate simulations^68^. Besides engineering the light focusing of metalenses, one can also take advantage of inverse design to better exploit other physical processes, such as optical^69^ nonlinear effects, and to gain better understanding of the multi-physics phenomena in photonic platforms. We believe that a more and more important role will be played by large-scale inverse-design methods in the future development of meta-optics.

## Methods

### Simulation

The meta-atoms are simulated using the method of RCWA. In the simulation setup, the height of the TiO_2_ meta-atoms is 600 nm, the periodicity of the unit cell is 400 nm, and the substrate is fused silica. The incident light is configured to LCP (RCP), and monitored light is in the opposite polarization state of RCP (LCP). The simulation wavelength sweeps from 480 nm to 680 nm in the visible.

### Fabrication

The metalenses are fabricated on glass wafers. The fabrication starts with spin-coating of resists in the following manner: a -thin-layer of Hexamethyldisilazane (HMDS), a layer of 600-nm-thick electron beam resist (Zeon Specialty Materials, ZEP-520A), and then a final layer of conductive polymer (Showa Denko, ESPACER 300) to dissipate charges during the following EBL process. After that, the 2-mm diameter metalens samples are exposed using Elionix ELS-F125 and the 1-cm diameter metalens is exposed using Elionix HS-50, respectively, followed by removal of the conductive polymer layer in water and development of exposed resist in o-Xylene solution, respectively. Next, a thin film of TiO_2_ is deposited onto the developed sample using low-temperature atomic layer deposition (Cambridge Nanotech, Savannah). TiO_2_ thin film is conformally deposited on the sample not only completely filling inside the developed area but also on top of the remaining resist film. The excessively grown TiO_2_ layer is later removed using reactive ion etching (Oxford Instruments, PlasmaPro 100 Cobra 300) with etchant gases of CHF_3_, O_2_, and Ar, until the underlying resist layer is exposed. In the final step, the resist layer is stripped off in solution of Remover PG (Kayaku Advanced Materials) at 85 °C for 24 hours, leaving only the TiO_2_ nanostructures on the glass wafer.

## Supplementary information

Supplementary information

Supplementary movie

## Data Availability

The meta-atom libraries that are used to design the metasurfaces in this study have been deposited in the Harvard University internal database. The data are available under restricted access for non-commercial use, and access can be obtained from the corresponding authors upon reasonable request.

## Code availability

The codes that support the findings of this study are available upon reasonable request from the corresponding authors.
