# Supplementary material for: Inverse design enables large-scale high-performance meta-optics reshaping virtual reality
Source: Nat Commun. 2022 May 3;13:2409. doi: 10.1038/s41467-022-29973-3 (PMC9064995; doi:10.1038/s41467-022-29973-3)
Supplement: Supplementary file 1 — Supplementary information [file 41467_2022_29973_MOESM1_ESM.pdf]

Supplementary Information

Inverse design enables large-scale high-performance meta-optics reshaping virtual reality

Zhaoyi Li[1†*], Raphaël Pestourie[2†], Joon-Suh Park[1,3], Yao-Wei Huang[1,4,5], Steven G. Johnson[2*], Federico Capasso[1*]

1. Harvard John A. Paulson School of Engineering and Applied Sciences, Harvard University, Cambridge, MA, USA.
2. Department of Mathematics, Massachusetts Institute of Technology, Cambridge, MA, USA.
3. Nanophotonics Research Center, Korea Institute of Science and Technology, Seoul, Republic of Korea.
4. Department of Electrical and Computer Engineering, National University of Singapore, Singapore.
5. Department of Photonics, National Yang Ming Chiao Tung University, Hsinchu, Taiwan.

† these authors contributed equally

* Corresponding emails: zhaoyili@seas.harvard.edu; stevenj@math.mit.edu; capasso@seas.harvard.edu.

Differentiable form of the optimization problem

The inverse design objective function: $\text{maximize}_{\mathbf{p}}\left(\min_{\lambda\in\lambda s}\left(I_\lambda(\mathbf{x}_{\mathbf{target}},\mathbf{p})\right)\right)$ as shown in the main text is non-differentiable. Fortunately, it is possible to reformulate the problem using an epigraph approach, where we optimize a "dummy" variable f_0 subject to the nonlinear constraints that it is smaller than the intensity at each of the wavelengths[1,2]

$$\text{maximize}_{\mathbf{p},f_0}(f_0)$$

$$\forall\lambda\in\lambda s, 0\geq\left(I_\lambda(\mathbf{x}_{\mathbf{target}},\mathbf{p})-f_0\right)$$

Given a function evaluating the nonlinear constraints and their gradients, a gradient-based optimization algorithm can find local optima in high-dimensional design space.[3] We used a multi-start approach to find a satisfying local optimum by exploring multiple random starting parameters.[4]

Principle of equivalence

The fields on a plane can be treated as equivalent current sources that generate the fields everywhere else. These equivalent electric (**J**) and magnetic (**K**) current densities are defined by the principle of equivalence:[5] $\begin{bmatrix}\mathbf{J}\\\mathbf{K}\end{bmatrix}=\begin{bmatrix}\hat{\boldsymbol{n}}\times\mathbf{H}\\-\hat{\boldsymbol{n}}\times\mathbf{E}\end{bmatrix}$ where $\hat{\mathbf{n}}$ is the surface unit-normal vector and the delta function implies that these are surface currents on the plane.

Implementation details of the approximate solver

The electric field at the target focal spot is computed using a convolution between the local sources and the appropriate Green's function

$$\vec{\mathbf{E}}(\vec{\mathbf{x}}_{\mathbf{target}}) = \int_{\Sigma} \vec{\mathbf{S}}_{\mathbf{local}}(\vec{\mathbf{x}}, \vec{\mathbf{p}}) \odot \overleftrightarrow{\mathbf{G}}(\vec{\mathbf{x}}, \vec{\mathbf{x}}_{\mathbf{target}}) d\vec{\mathbf{x}}$$

The support of the integral is technically infinite, but a "windowing" method truncates this integral accurately to a finite region.[6] This integral is two-dimensional, which is expensive to evaluate for large area metasurfaces (diameter > 1000λ). To improve efficiency, we impose cylindrical symmetry in the parameter function, which translates into a radial local field and a radial source function. Even though the meta-atom parameters are chosen to be rotationally symmetrical, the surface is not axisymmetric at a subwavelength scale, in contrast to recent work on axisymmetric metasurfaces.[7] In our locally periodic approximation, the Green's function is also cylindrical symmetric when the focal spot is on the focal axis, so the problem is reduced to the one-dimensional convolution, which dramatically speeds up the evaluation of the intensity $I(\mathbf{x}_{\mathbf{target}}) = \left|\vec{\mathbf{E}}(\mathbf{x}_{\mathbf{target}})\right|^{\mathbf{2}}$ in the far field. A surrogate model—a data-driven fit that predicts the solution of Maxwell's equations instead of solving the equations numerically during optimization—is used to evaluate the local field of a given set of parameters efficiently ($10^6\times$ speed gain, see "Surrogate model vs. RCWA" section below). Here, the surrogate model is based on Chebyshev interpolation.

Equivalence principle and Green's function for polarization conversion

First, we transpose the mode on the Cartesian basis, where the dyadic Green's function is well known. With the focal axis and the propagation along the z-direction, the right circular polarization (RCP) corresponds to $\frac{E_x\hat{\mathbf{x}}+iE_y\hat{\mathbf{y}}}{\sqrt{2}}$ in the Cartesian basis $\hat{\mathbf{x}}$ and $\hat{\mathbf{y}}$ (for a wave propagating in the z-direction), where $E_x = E_y = E_{RCP}$ is the complex coefficient of the RCP mode. The principle of equivalence transforms an electric field into an artificial magnetic current $-\hat{\mathbf{n}} \times \mathbf{E} = \mathbf{K}$ where $\hat{\mathbf{n}} = \hat{\mathbf{z}}$ is the normal to the metasurface [5]. Therefore $\mathbf{K} = -\hat{\mathbf{z}} \times \frac{E_x\hat{\mathbf{x}}+iE_y\hat{\mathbf{y}}}{\sqrt{2}} = \frac{iE_y}{\sqrt{2}}\hat{\mathbf{x}} - \frac{E_x}{\sqrt{2}}\hat{\mathbf{y}}$, the equivalent currents are $\mathbf{K_x} = \frac{i\mathbf{E_y}}{\sqrt{2}}$ and $\mathbf{K_y} = -\frac{\mathbf{E_x}}{\sqrt{2}}$. The non-zero Green's functions in the vacuum for these two currents are $G_{KxEy}(\mathbf{x_1}, \mathbf{x_2}) = -f(r)\hat{\mathbf{r}} \times \hat{\mathbf{x}} \cdot \hat{\mathbf{y}} = -f(r)r_z$ and $G_{KyEx}(\mathbf{x_1}, \mathbf{x_2}) = -f(r)\hat{\mathbf{r}} \times \hat{\mathbf{y}} \cdot \hat{\mathbf{x}} = f(r)r_z$, respectively. Here, r_z is the z-component of the normalized vector $\hat{\mathbf{r}} = \frac{\mathbf{r}}{r}$ where $\mathbf{r} = \mathbf{x_1} - \boldsymbol{x_2}$, and $r = |\mathbf{r}|$. The coefficient is a radial function $f(r) = \frac{nk}{4\pi r}\exp\left(i\left(kr + \frac{\pi}{2}\right)\right) Z\left(1 - \frac{1}{ikr}\right)$, where $Z = \sqrt{\frac{\mu}{\epsilon}}$ is the impedance of free space, $n = \sqrt{\epsilon\mu}$, and $k = \frac{2\pi n}{\lambda}$. r_z is constant when $\mathbf{x_2}$ is on the cylinder axis as we swipe the rotation angle ϕ of the polar coordinate $\mathbf{x_1}$ and fix its radius, so the Green's function is cylindrical symmetric when the focal spot is on the focal axis of the lens. The contribution of a given radius *r* to the far-field is then $\int \left(\mathbf{K_y}G_{KyEx}((r,\phi), x_2)\hat{\mathbf{x}} + \mathbf{K_x}G_{KxEy}((r,\phi), x_2)\hat{\mathbf{y}}\right) d\phi = \int \left(-\frac{\mathbf{E_x}}{\sqrt{2}}f(r)r_z\hat{\mathbf{x}} - \frac{i\mathbf{E_y}}{\sqrt{2}}f(r)r_z\hat{\mathbf{y}}\right) d\phi = -\frac{2\pi}{\sqrt{2}}E_{RCP}f(r)r_z(\hat{\mathbf{x}} + i\hat{\mathbf{y}})$ where (r, ϕ) is the position of the source in polar

coordinates, which shows that the convolution boils down to a single radial term in the integrand. The reasoning is similar for a left circular polarization.

Wirtinger calculus and gradient of the intensity at the focal spot

Wirtinger calculus[8] is a common framework to differentiate complex-valued functions. Instead of taking the partial derivative of a complex number $z = a + ib$ with respect to its real and imaginary parts, Wirtinger calculus takes the partial derivatives with respect to z and its complex conjugate $\bar{z} = a - ib$. This formalism is especially useful for non-holomorphic functions of the complex plane like $f(z) = |z|^2 = z\bar{z}$, where the differential becomes $df = \frac{\partial f}{\partial z}dz + \frac{\partial f}{\partial \bar{z}}d\bar{z} = \bar{z}dz + zd\bar{z} = 2\Re e(\bar{z}dz)$.

In the case of the gradient of the intensity, the relevant complex number is the electric field **E** and the intensity is $I(\mathbf{p}) = \mathbf{E}(\mathbf{p})\bar{\mathbf{E}}(\mathbf{p})$, where **p** is the vector of all the parameters defining the metasurface.

Using the differential from Wirtinger calculus, we get dI(**p**)= $2\Re e(\bar{\mathbf{E}}(\mathbf{p})d\mathbf{E}(\mathbf{p}))$. From the differential, we can define the gradient with respect to the metasurface parameters $\nabla_{\mathbf{p}}I(\mathbf{p}) = 2\Re e\left(\bar{\mathbf{E}}(\mathbf{p})\nabla_{\mathbf{p}}\mathbf{E}(\mathbf{p})\right)$.

The only thing left to do is to find the gradient $\nabla_{\mathbf{p}}\mathbf{E}(\mathbf{p})$, starting from Equation. 1 in the main text

$$\vec{\mathbf{E}}\left(\mathbf{x}_{\mathbf{target}}\right) = \int_{\Sigma} \vec{\mathbf{S}}_{\mathbf{local}}(\mathbf{x},\ \vec{\mathbf{p}}) \odot \overleftrightarrow{\mathbf{G}}\left(\mathbf{x},\ \mathbf{x}_{\mathbf{target}}\right)d\mathbf{x}$$

We can transfer $\nabla_{\mathbf{p}}$ inside the integral sign using the dominated convergence theorem

$$\nabla_{\mathbf{p}}\ \vec{\mathbf{E}}\left(\mathbf{x}_{\mathbf{target}}\right) = \int_{\Sigma} \nabla_{\mathbf{p}}\ \left(\vec{\mathbf{S}}_{\mathbf{local}}(\mathbf{x},\ \vec{\mathbf{p}}) \odot \overleftrightarrow{\mathbf{G}}\left(\mathbf{x},\ \mathbf{x}_{\mathbf{target}}\right)\right) d\mathbf{x}$$

Since the Green's function is independent of the parameters of the metasurface, it can be factored out of the differentiation.

$$\nabla_{\mathbf{p}}\vec{\mathbf{E}}\left(\mathbf{x}_{\mathbf{target}}\right) = \int_{\Sigma} \nabla_{\mathbf{p}}\vec{\mathbf{S}}_{\mathbf{local}}(\mathbf{x},\ \vec{\mathbf{p}}) \odot \overleftrightarrow{\mathbf{G}}\left(\mathbf{x},\ \mathbf{x}_{\mathbf{target}}\right)d\mathbf{x}$$

For completeness, $\nabla_{\mathbf{p}}\vec{\mathbf{S}}_{\mathbf{local}}(\mathbf{x},\ \vec{\mathbf{p}})$ is obtained by taking the analytical derivative of the surrogate model for the local field, i.e., the derivative of our Chebyshev interpolation of the zeroth order Fourier coefficient of the transmitted field with respect to the meta-atom parameter. Therefore, the surrogate model enables us to efficiently evaluate both the local field and its gradient. In contrast, methods that are not based on a surrogate model need to solve for Maxwell's equation both for the forward and the adjoint problem.

Cylindrical symmetry and Cartesian grid

To further speed up the inverse design, we use a solver with cylindrical symmetry. The parameter function we use is cylindrical symmetric; however, the evaluation on the parameter function is on a Cartesian grid.

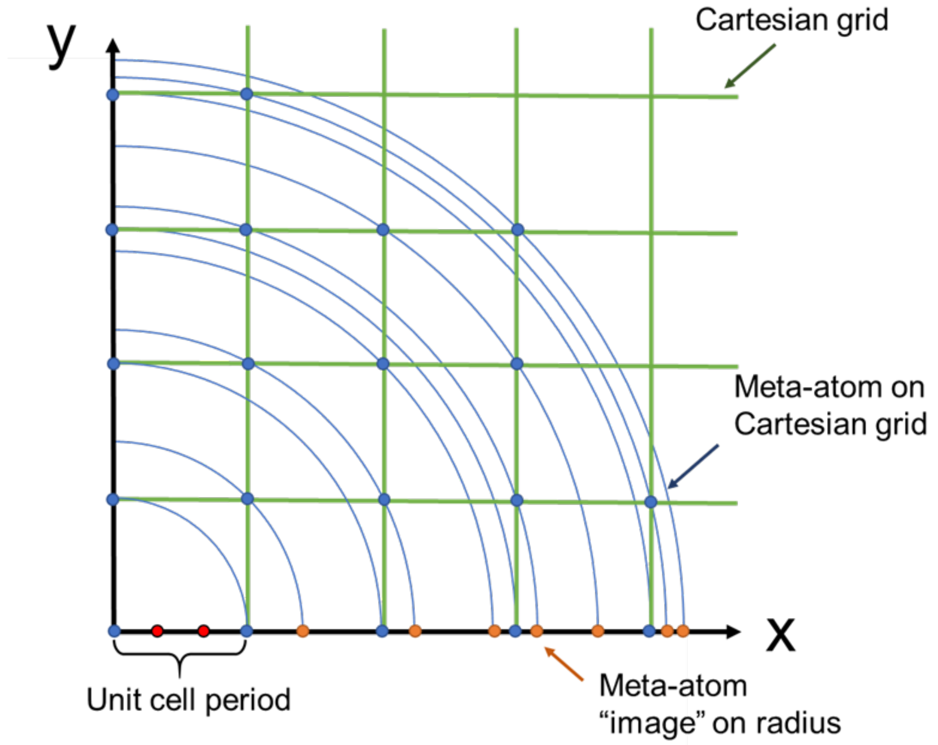

Figure S1. When defining the parameters on a Cartesian grid (blue dots) using a cylindrically symmetry function, the number of degrees of freedom (orange dots) is significantly bigger than the Cartesian grid resolution (green lines).

Figure S1 shows the schematic of a metalens, which has meta-atom (blue dots) arranged in Cartesian coordinates (green grid). The spacing between the grid is the unit cell period. Each meta-atom has an “image” on radial axis (X) as represented by an orange dot. When imposing cylindrical symmetry, we consider the contribute from each meta-atom on the Cartesian grid via its “image” along the radial direction. In simulation, we have two additional sampling points within each unit cell period shown as red dots. The EM responses of the “image” meta-atoms (orange dots) are estimated through interpolation using the fine grid.

Surrogate model vs. RCWA

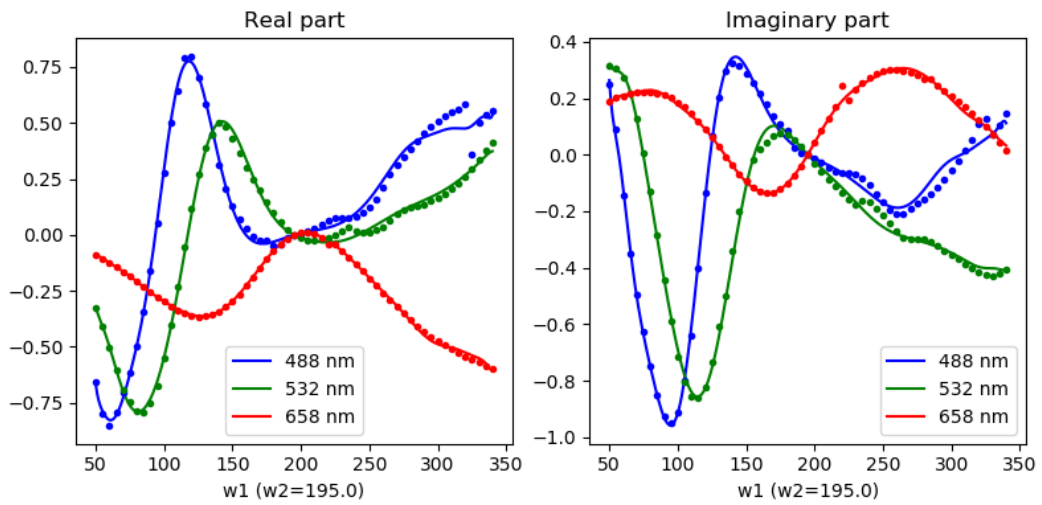

Figure S2. Surrogate model against simulated data. The interpolation results from Chebyshev regression are smoother than the real data (evaluated on a grid with resolution of 5nm with lower bound of 50 and upper bound of 340, 3481 simulations). The smoothing is obtained by fitting a polynomial on 3481 points using polynomials of up to order 22 (250 polynomials), done on purpose to avoid big oscillations in the interpolation from resonance in the data. The error from the smoothing is negligible compared to the error from LPA or fabrication errors.

In contrast to a solver, which provides the full electric and magnetic fields when given a discretized geometry, a surrogate solver returns the complex transmission when given a parameterization of the geometry. To train our surrogate model, the data was rigorously simulated on a grid with the width and the length ranging from 50 to 340 by increment of 5 nm using an RCWA method based on reticolo,[9] which results in 3481 simulations. Chebyshev regression fits a surrogate with 250 polynomials of degree up to 22, using the Chebyshev basis. The implementation uses ApproxFun.jl in Julia language, and the regression is performed by constructing the Chebyshev–Vandermonde matrix and performing a least-squares fit. Chebyshev regression smooths the data, especially where there are resonances, to reduce rapid oscillations in the surrogate model (which may result in poor local optima). The error introduced by this smoothing is negligible compared to the error of LPA or fabrication error, because the optimal design largely employs parameters far from such resonances. Evaluation of the surrogate model takes 8.5 µs on average, whereas solving with the RCWA library takes 10 s on average, which corresponds to a **6 orders-of-magnitude** speedup for our surrogate-based model.

The experimental validation matches numerical results because the focal lengths measured correspond to the ones optimized by the inverse-design framework. This shows that LPA is accurate in the regime of our lenses' designs. Various computational methods to improve upon the accuracy of LPA exist, including overlapping-domain decomposition,[10] or spatially truncated multipole expansions[11] (which unfortunately cannot currently incorporate substrates). However, in the regime where LPA works, as in the case of this paper, the more accurate solver gives very similar results to LPA but at a major sacrifice in performance (especially when surrogates are used in LPA). LPA is quite robust to differences in neighboring atoms, and is observed to work well even for large variations in localized portions of the metasurface—it only breaks down in

situations very different from those in this paper involving rapid variations over large areas, such as occur in designs for very oblique angles of incidence[12] or for very high numerical apertures.[13]

Optical setup for metalens measurements

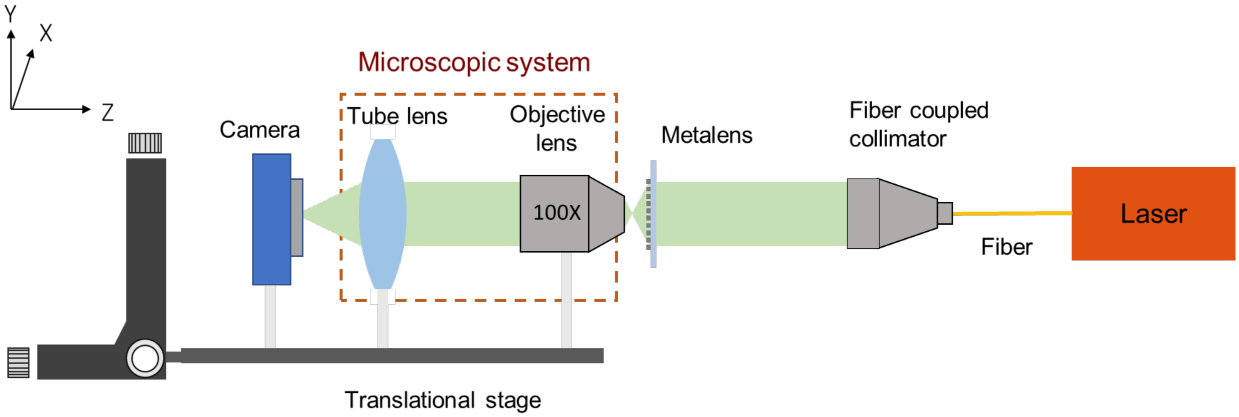

Figure S3. Schematic of the optical setup used to measure the focusing performance of the metalens in three dimensions. The laser output (either from laser diodes or a super continuum laser) is collimated by a fiber coupled collimator (Thorlabs, RC12APC-P01) and incident on the metalens. The three-dimensional intensity distribution of the focal spot is then imaged through a home-built microscopic system as shown in the dashed box. It consists of a 100X objective lens (Olympus), a tube lens (Thorlabs TTL180-A), and a CMOS camera (Thorlabs, DCC1545M). The microscopic system is mounted on a translational stage.

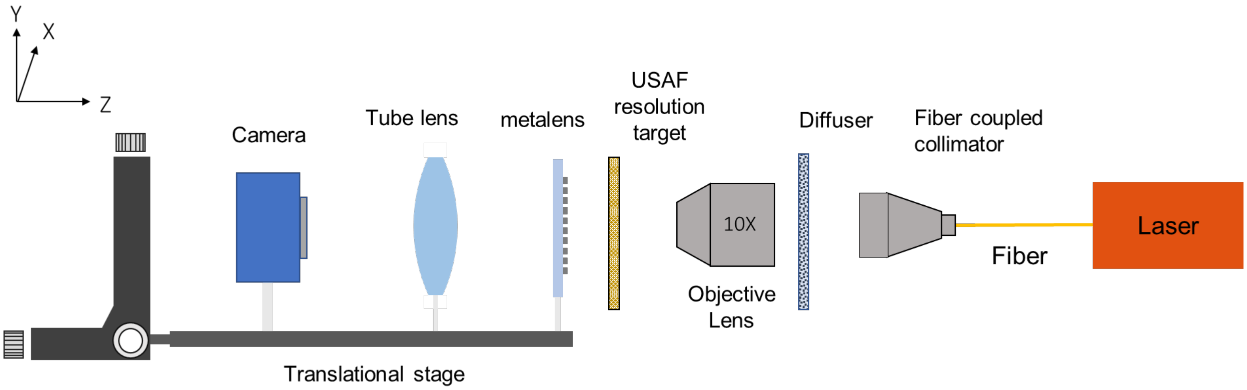

Figure S4. Schematic of the optical setup used to characterize the imaging performance of the metalens. The laser source is either a laser diode or a super continuum laser. The output light is collimated by a fiber coupled collimator (Thorlabs, RC12APC-P01) and goes through a diffuser. It is then slightly focused by an objective lens (Mitutoyo objective, 10x magnification) onto the United States Air Force resolution target. The metalens is placed at focal length away from the surface of the target. The relayed image by the metalens and the tube lens (Thorlabs TTL180-A) is captured by a CMOS camera (PCO, panda 4.2). The metalens, the tube lens, and the camera are mounted on a translational stage.

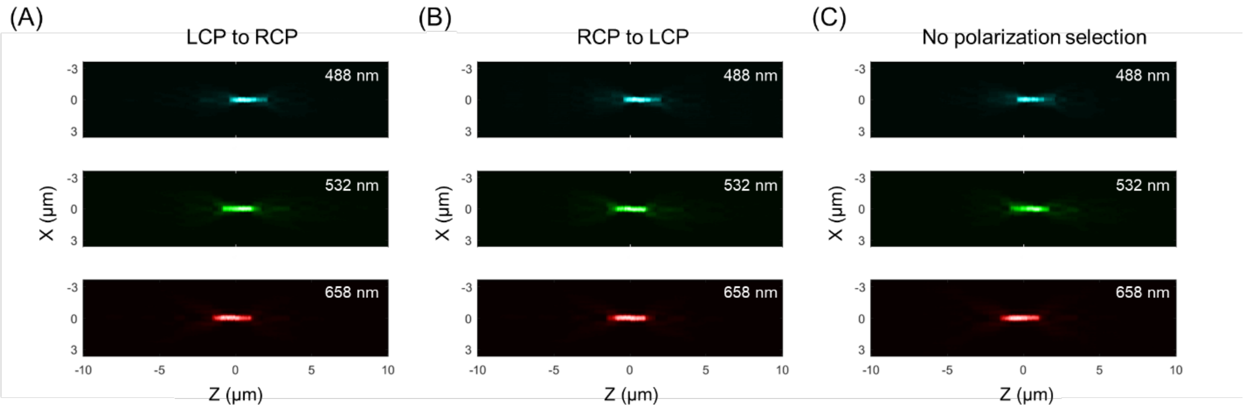

Figure S5. Measured focal intensity distribution in the XZ cross section at design wavelengths when (A) incident light is in the LCP state, and output light is selected in the RCP state; (B) incident light is in the RCP state, and output light is selected in the LCP state; (C) no particular polarization state is selected for incident and output light.

We measured the focusing intensity distribution of the 2-mm-diameter, NA = 0.7, RGB-achromatic metalens in three polarization configurations. We put a polarizer pair consisting of a wire grid polarizer and an achromatic quarter waveplate between the collimator and metalens to select the polarization state of incident beam, and we put another analyzer pair between the tube lens and the CMOS camera to select the polarization state of output beam. Figure S5(A) shows the measured focusing intensity distribution in the XZ cross section when we configure the incident light in the LCP state and output light in the RCP state. Figure S5(B) shows the measurement result when we configure the incident light in the RCP state and output light in the LCP state. The measurement results in two configurations are identical. It is because each meta-atom has the same response when converting light from an LCP state to an RCP state or vice versa according to Jones' matrix. Since any polarization state can be decomposed into the orthogonal basis of LCP and RCP states, the metalens can work for incidence in an arbitrary polarization state and has polarization-insensitive focusing performance. Figure S5(C) (as also shown in the main text) shows the measurement results when we did not particularly select the polarization state of incident or output light (no polarizer and analyzer is used), and it shows the same focusing performance as Fig. S5(A) and (B).

Point spread functions (PSFs) of the RGB-achromatic metalens

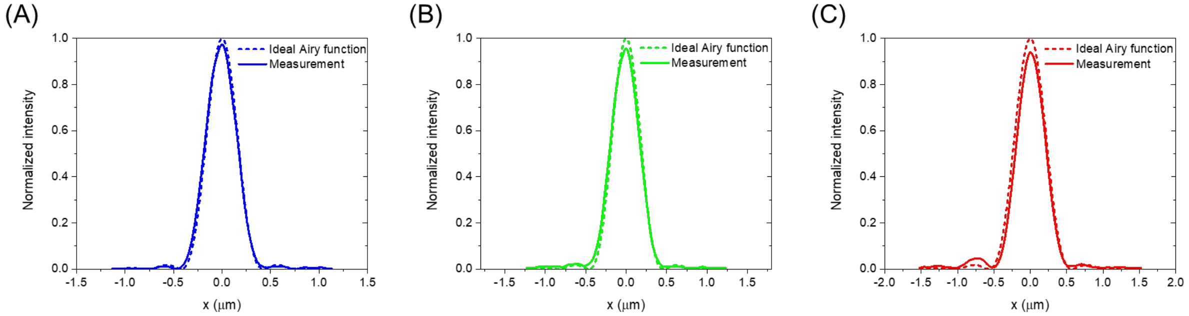

Figure S6. Measured focal intensity line profile (solid line (A) to (C)) at 488 nm, 532 nm, and 658 nm, respectively, in comparison with ideal Airy function profiles (dashed line).

Figure S6 shows the focal intensity line profile of the RGB-achromatic metalens (NA = 0.7) at blue, green, and red wavelengths. The theoretical Airy function profiles are also shown for comparison. The calculated Strehl ratios are 0.97, 0.96, and 0.94, respectively. Here, the light sources used are three laser diodes.

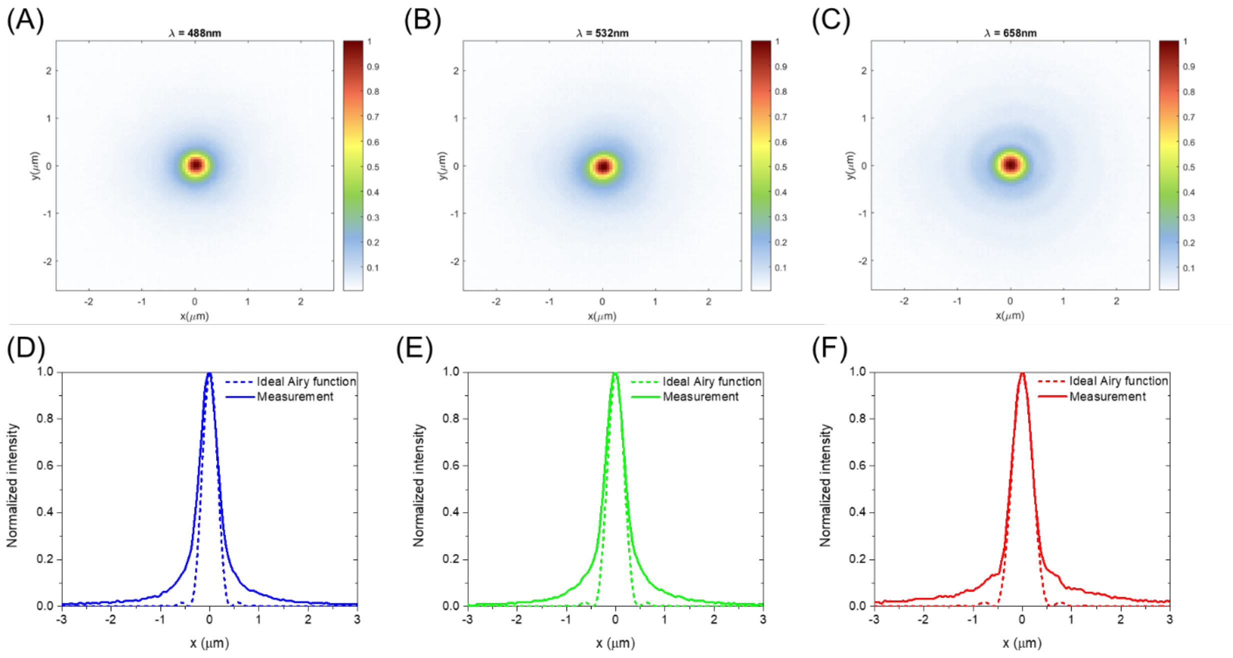

Figure S7. Top ((A) to (C)): Measured focal intensity distribution in the XY cross section at 488 nm, 532 nm, and 658 nm, respectively, using super continuum laser as the light source instead of laser diodes. Bottom ((D) to (F)): Measured focal intensity line distribution (solid line) in comparison with their respective ideal Airy function profiles (dashed line).

In comparison, we also used a super continuum laser as the light source to measure the focal spots of the metalens. Figure S7, (A) to (C), shows the focal intensity distribution in the XY cross section at design wavelengths. Figure S7, (D) to (F), shows the corresponding focal intensity line distribution. The measurement shows slightly different from the previous measurement when the laser diodes are used. There are some background light around focal spots, and the PSF profile shows focusing intensity tail decaying as radial distance in contrast with Airy function profile, which shows negligible focusing intensity within side lobes.

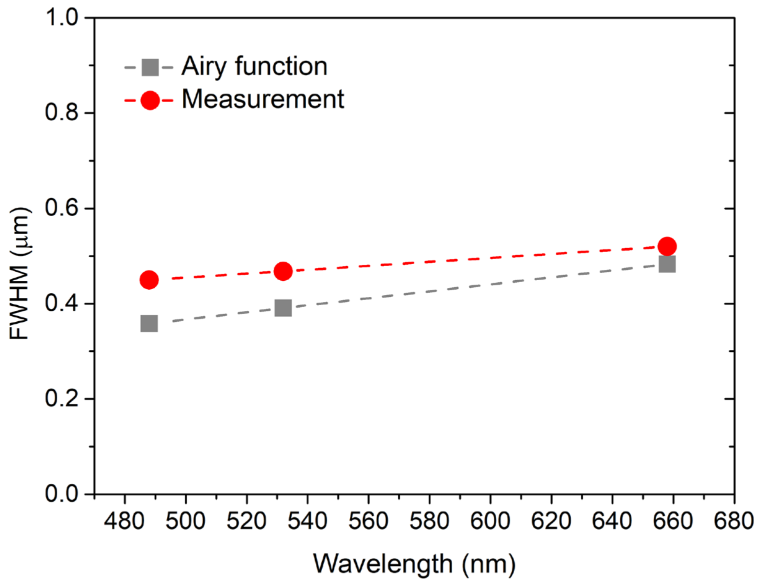

Figure S8. Measured full width half maxima (FWHMs) at design wavelengths, which are compared with FWHMs of ideal Airy functions.

The measured FWHMs at wavelengths of 488 nm, 532 nm, and 658 nm are 450 nm, 468 nm, and 520 nm, respectively as shown in Fig. S8. They are slightly larger than the theoretical values of Airy functions, which are 358 nm, 391 nm, and 483 nm, respectively.

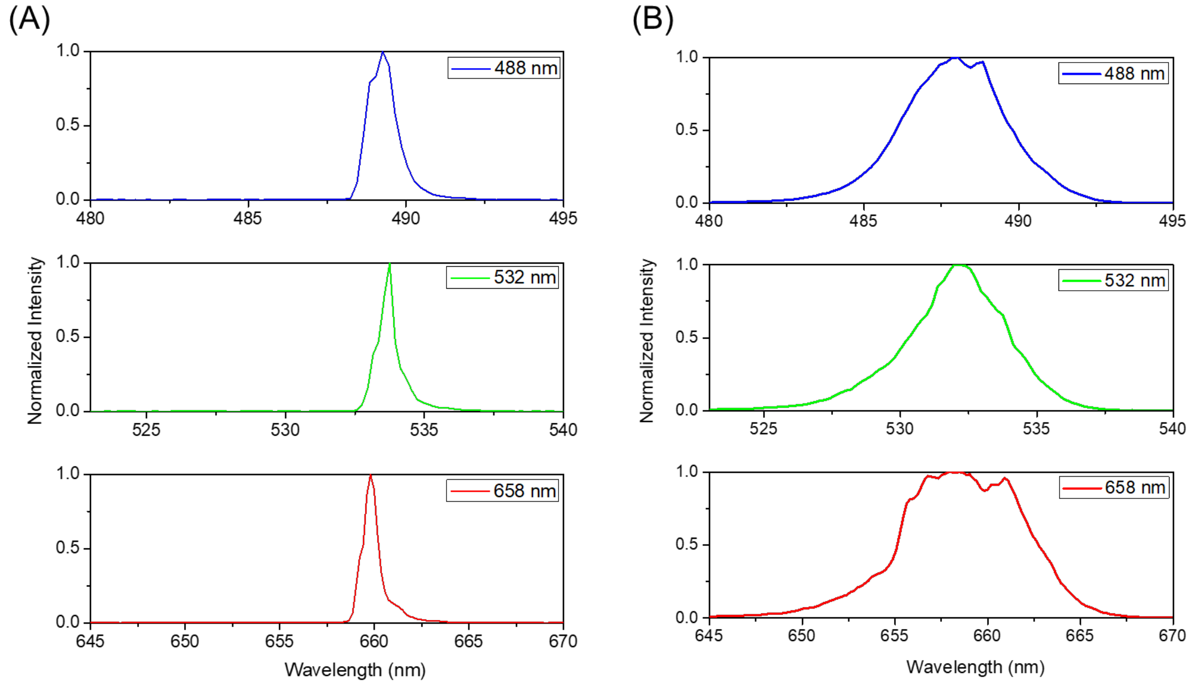

Figure S9 (A). Measured spectra of blue, green, and red laser diodes. (B). Measured spectra of a super continuum laser when the peak intensity of output light is tuned to center at 488 nm, 532 nm, and 658 nm (corresponding to operation wavelengths of laser diodes).

The measurement difference between two experiments can be explained by the laser spectra. Figure S9(A) shows the spectra of the laser diodes operating at blue, green, and red wavelength. In comparison, Figure S9(B) shows the spectra of the super continuum laser when the wavelength of the peak intensity is tuned to match with laser diodes. The laser diodes have a linewidth of 0.5 – 1 nm whereas the super continuum laser has a minimum linewidth of 4 – 7 nm, which is about one order of magnitude larger. The larger beam linewidth of the super continuum laser causes the broadening of the focal spots and the showing up of the focal intensity tails.

Focusing near the design wavelength

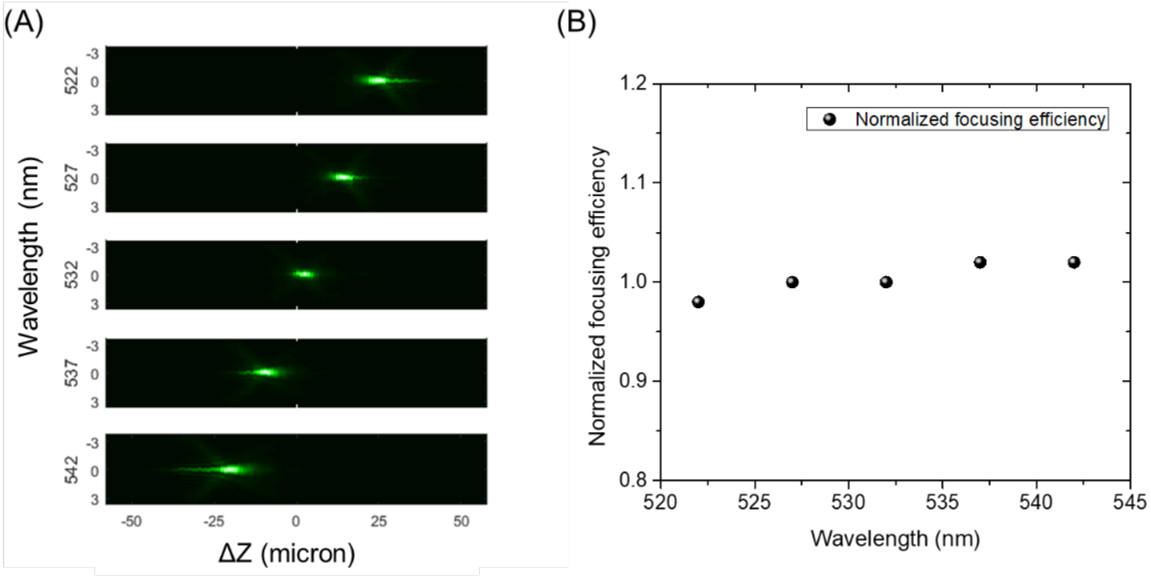

Figure S10 (A) Measured focusing intensity distribution of the 2-mm-diameter RGB-achromatic metalens in the XZ plane near the design wavelength of 532 nm. (B) Measured normalized focusing intensities. The focusing efficiency is normalized to the one at 532 nm.

The measured focusing intensity distribution near the design wavelength of 532 nm for the 2-mm-diameter RGB-achromatic metalens is shown in Fig. S10(A). The metalens is chromatic near the design wavelength as can be seen from the focal shift. Within a 20nm bandwidth, the light is well focused. The measured focusing intensity, normalized to the one at 532 nm, is also shown in Fig. S10(B). It is possible to not only realize multi-wavelength-achromatic focusing but also to alter the dispersion near the design wavelength by using a hybrid lens system that combines a metasurface with a refractive lens.[14]

Additional USAF imaging results by the RGB-achromatic metalens (NA = 0.7)

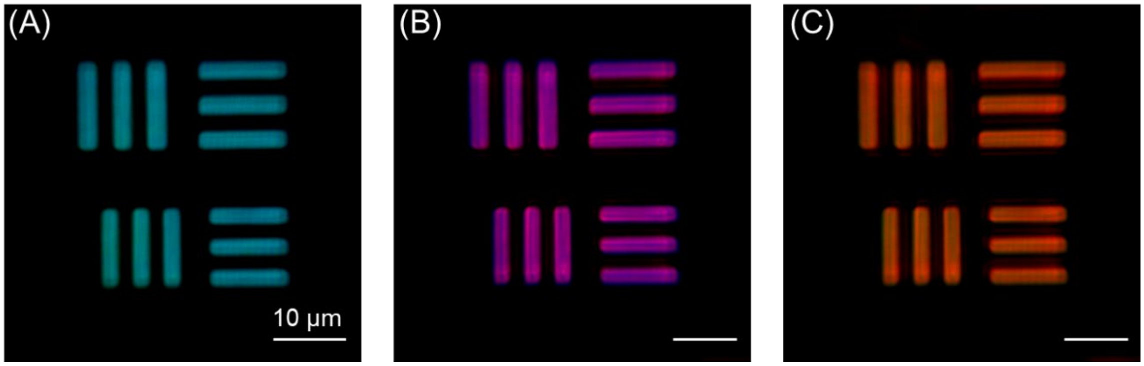

Figure S11. Imaging results of the USAF resolution target group No.7 elements No.5 and 6. under synthesized color illumination.

Figure S11 shows additional imaging results of the elements No.5 and 6 from the group No.7 of the USAF resolution target by the RGB-achromatic metalens. The smallest linewidth is 2.2 µm. The illumination color is synthesized by mixing blue and green light (A), blue and red light (B), and green and red light (C). The imaging results under synthesized illumination prove the RGB-achromatic imaging performance of the metalens. As long as the metalens has diffraction-limited and achromatic imaging performance for primary RGB colors, it will have the same imaging performance for other synthesized colors. It implies a platform for display imaging.

Focusing measurement of the polychromatic metalenses

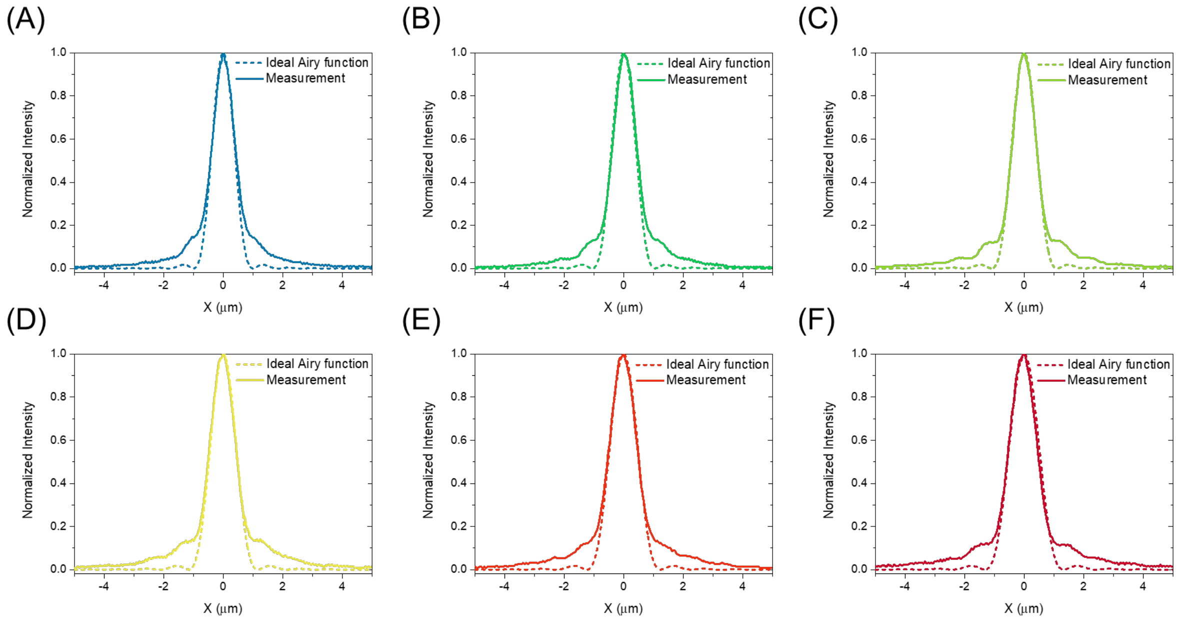

Figure S12 (A) to (F). Measured focal intensity line distribution of the six-wavelength-achromatic metalens with NA = 0.3 at design wavelength of 490 nm, 520 nm, 540 nm, 570 nm, 610 nm, and 650 nm, respectively. The solid lines are the measurements, and the dashed lines are the theoretical Airy function profiles.

Figure S12 shows the measured focal intensity line profile of the polychromatic metalens with NA = 0.3 at six design wavelengths. The Airy function profiles are also shown in dashed lines. The discrepancy between the measurements and the Airy function profiles, which shows up at the focal intensity peak tails, is due to the large linewidth of the super continuum laser.

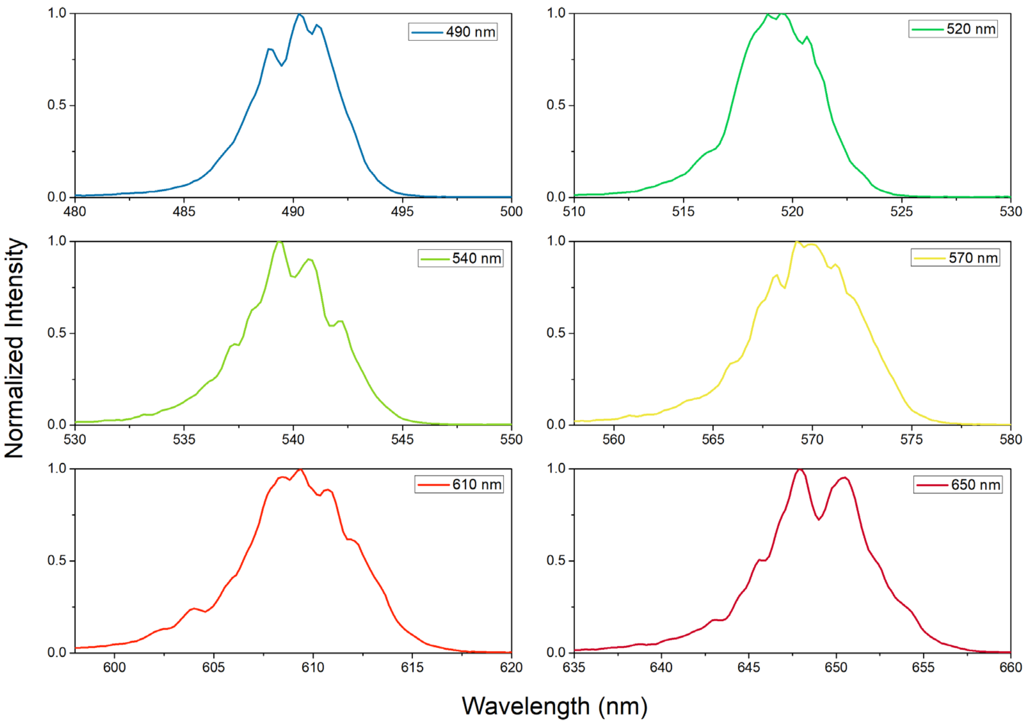

Figure S13. Measured spectra of the super continuum laser when the output light intensity peak is tuned to center at six wavelengths.

The measured linewidths of the supercontinuum laser are summarized in Fig. S13. Here, the supercontinuum laser is tuned to center the peak intensity at wavelengths of 490 nm, 520 nm, 540 nm, 570 nm, 610 nm, and 650 nm, respectively. The linewidth (FWHM of the spectrum) ranges from 4.5 nm to 7 nm.

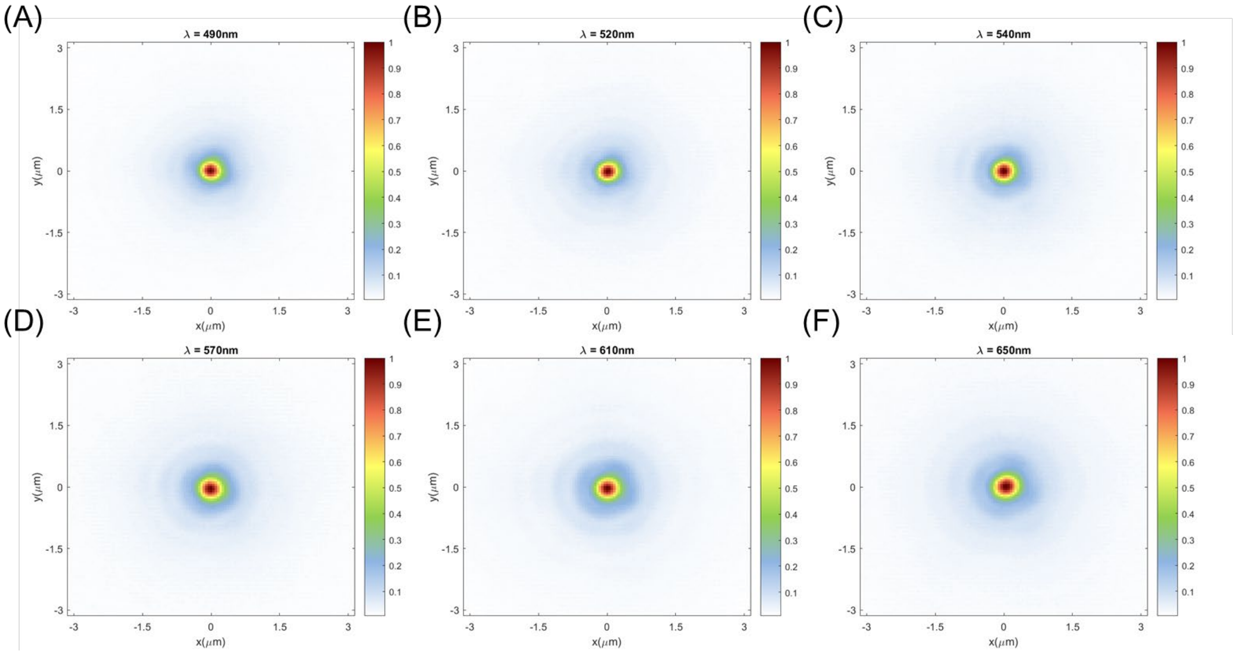

Figure S14. Measured focal intensity distribution of the six-wavelength-achromatic metalens with NA = 0.7 at design wavelengths of 490 nm, 520 nm, 540 nm, 570 nm, 610 nm, and 650 nm, respectively.

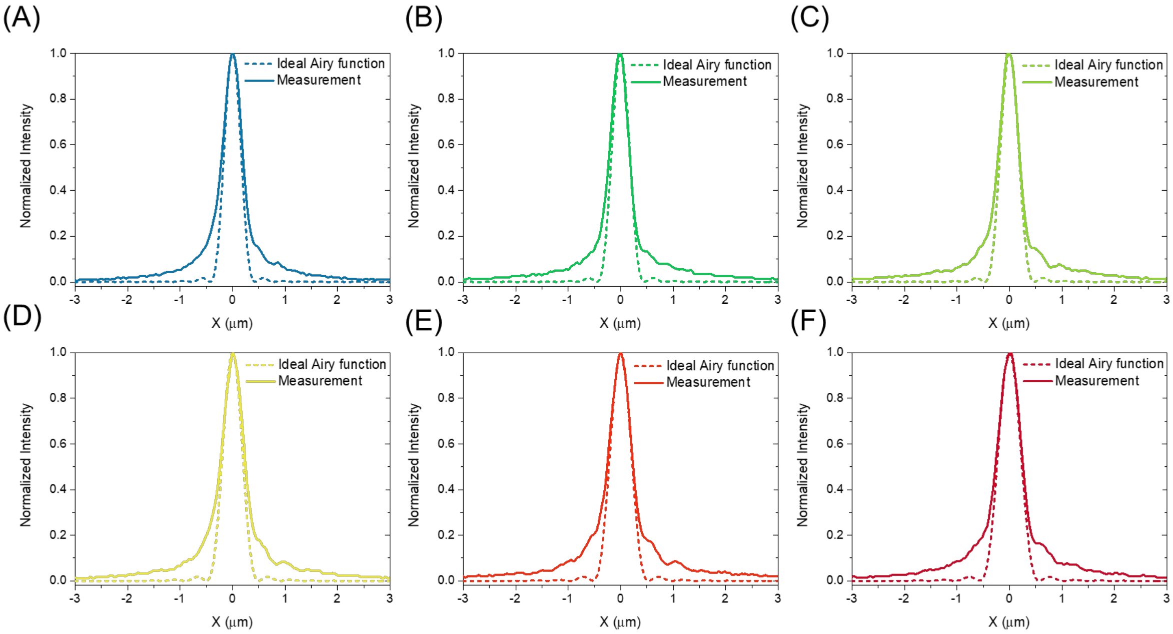

Figure S15. Measurement of focal intensity line profiles (solid lines) at design wavelengths in comparison with the theoretical Airy function profiles (dashed lines).

focal intensity line profile along radial direction, which has a good agreement with Airy function profiles. The measured FWHMs are shown in the main text.

Focusing efficiency of the polychromatic metalens

	Polychromatic metalens (2 mm; NA = 0.3)					
Wavelength	490 nm	520 nm	540 nm	570 nm	610 nm	650 nm
Focusing efficiency	8.7%	7.8%	8.3%	8.2%	8.1%	7.2%

Table S1. Measured focusing efficiency of a 2-mm-diameter six-wavelength-polychromatic metalens at design wavelengths.

USAF imaging results by the polychromatic metalens (NA = 0.3)

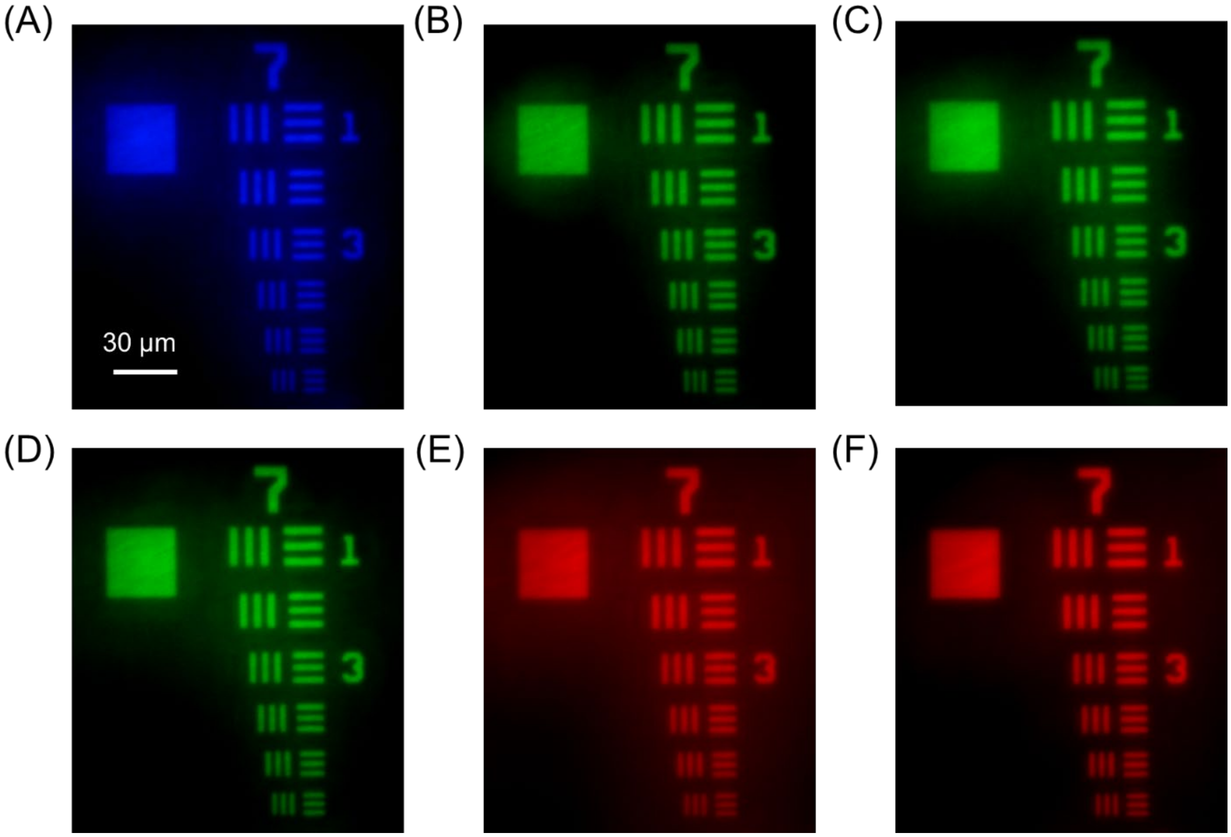

Figure S16. Imaging results of the USAF resolution target by the polychromatic metalens (NA = 0.3) at wavelength of (a) 490 nm (b) 520 nm (c) 540 nm (d) 570 nm (e) 610 nm (f) 650 nm.

Point spread functions of the cm-scale RGB-achromatic metalens

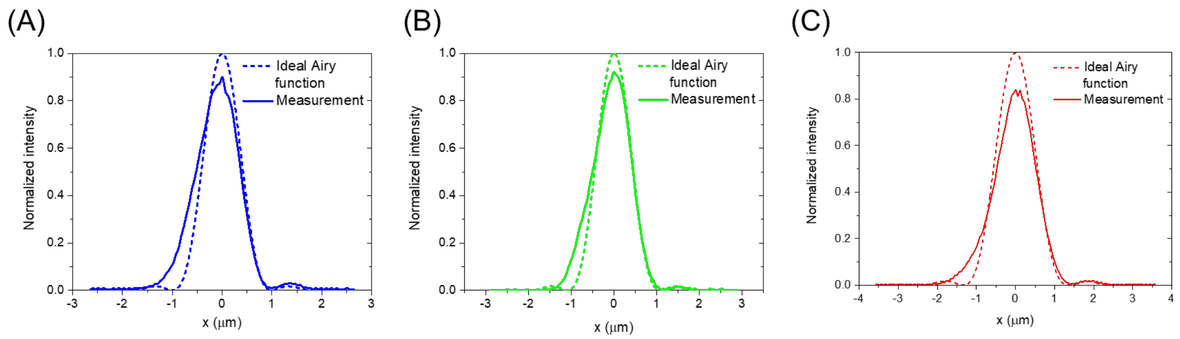

Figure S17 (A) to (C). The solid lines are measured focal intensity distribution line profiles of the cm-diameter, RCB-achromatic, NA = 0.3 metalens at design wavelengths of 488 nm, 532 nm, and 658 nm. The dashed lines are their respective Airy function profiles.

The focal intensity line distribution of the cm-scale RGB-achromatic metalens at design wavelengths of 488 nm, 532 nm, and 658 nm is summarized in Fig. S17, (A) to (C), respectively. The Airy function profiles of NA = 0.3 are shown in the dashed lines. The calculated Strehl ratios are 0.90, 0.92, and 0.84, respectively. The light illuminance over the 1cm aperture area is not uniform, thus the measured focal profiles are slightly asymmetric.

	RGB-achromatic metalens (1cm)		
Wavelength	488 nm	532 nm	658 nm
Focusing efficieny	16.1%	13.6%	15.8%

Table S2. Measured focusing efficiency of the 1-cm-diameter RGB-achromatic metalens at design wavelengths.

Forward design vs. Inverse design

We used our 1cm-diameter RGB-achromatic metalens design as a benchmark to compare forward design methods with our inverse design method. The results are summarized in the following Table.S3 including the objective functions and simulation results of focusing efficiencies at design wavelengths.

	Forward design			
	Objective function	488 nm	532 nm	658 nm
1	$\min\left(\sum_{1}^{n} \Delta\varphi_i^{\,2}\right)$	1.2%	1.1%	2.5%
2	$\min\left(\frac{\sum_1^n \Delta\varphi_i^{\,2}}{\sum_1^n T_i}\right)$	13.3%	9.4%	22.0%
3	$\min\left(\sum_{1}^{n} \frac{\Delta\varphi_i^{\,2}}{T_i}\right)$	15.3%	10.1%	20.8%
4	$\min\left(\frac{\max\left(\Delta\varphi_i^{\,2}\right)}{\sum_1^n T_i}\right)$	12.6%	13.3%	18.5%
	**Inverse design**			
	$max\left(\min_{\lambda\in\lambda_s}(I_\lambda(\vec{\boldsymbol{x}}_{target}, \vec{\boldsymbol{p}}))\right)$	**23.8%**	**23.8%**	**23.8%**

Table S3. Comparison of forward design and inverse design strategies: optimized focusing efficiencies of 1cm-diameter RGB-achromatic polarization-insensitive metalens.

Here, $\Delta\varphi_i$ is the phase difference between a design phase and a realized phase by a meta atom at ith wavelength: i.e., $\Delta\varphi_i = mod(\varphi_{design} - \varphi_{meta}, 2\pi)$ and is in the range of -π to π. The math operator mod denotes the remainder after division. T_i is the normalized transmission coefficient of a meta atom at i th wavelength. For a fair comparison, we use the same meta library for both methods. In a forward design, a proper meta atom is chosen from the meta atom library to be placed at each lens position according to an objective function. Each atom is optimized independently. The objective function #1 considers only phase matching conditions and shows only 1~2% focusing efficiency at three design wavelengths. This is because the transmission of a meta atom is not uniform at the design wavelengths, and a good phase matching is realized at the cost of low and non-uniform transmission. The objective functions of forward designs #2 - #4 contain not only the phase matching condition but also the transmission. They aim to decrease the phase errors and increase the transmission of meta atoms simultaneously. The corresponding results show significant improvements compared to using objective function #1. The highest optimal focusing efficiency is 22% at wavelength of 658 nm. However, the optimization results vary with the definition of the objective functions, and the optimized focusing efficiencies are not uniform at RGB wavelengths. The above optimization results reveal the challenges when using a forward design to solve a design problem that involves multiple goals and is subject to multiple constraints. In this case, the forward design results in lower and non-uniform focusing efficiencies at achromatic wavelengths due to the lack of systematic feedback loops for optimization (in another word, it is a one-way design).

In comparison, our inverse design method achieves uniform 24% focusing efficiencies at RGB wavelengths. The meta atoms are optimized during iterations according to the feedbacks of objective function evaluation that directly reflects the performance of a desired functionality.

Display optical setup and strategies for improvement

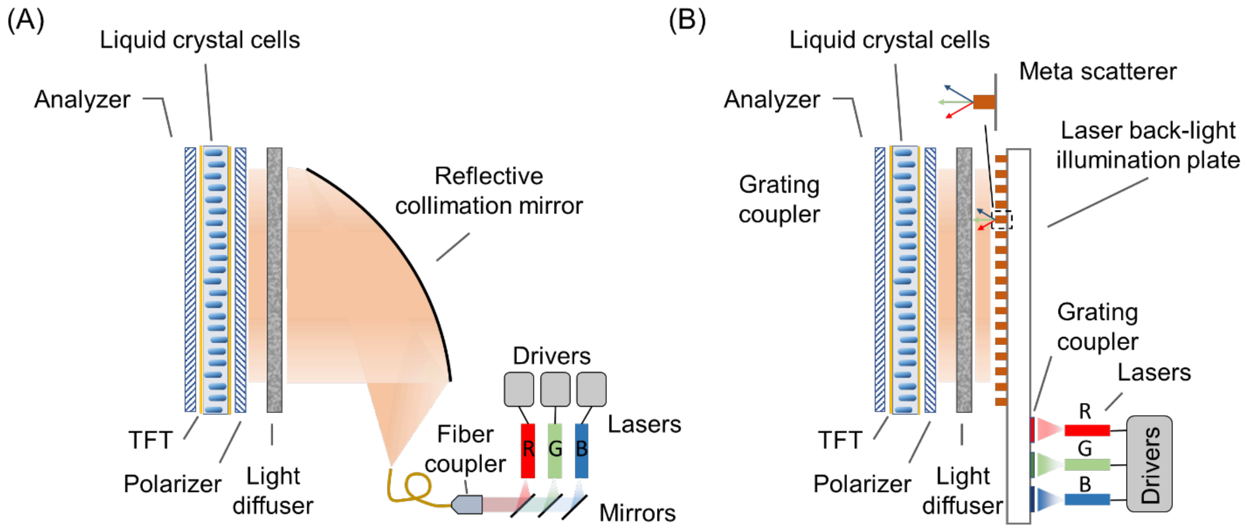

Figure S18 (A) Our current optical setup for the laser back-illuminated micro-LCD system. (B) Our proposed optical setup for a compact display system, where a waveguide-based illumination plate is employed.

In our current laser back-illuminated micro-LCD setup as shown in Fig. S18(A), we use red, green, and blue laser diodes as the RGB illumination sources. The RGB laser output beams are combined and coupled into a common fiber coupler after reflection and alignment through mirrors. The light emitted out from a fiber tip is then reflected and collimated by a reflective collimation mirror. The collimated RGB laser light goes through a light diffuser and is then incident onto the display panel. The form factor of the laser back-illuminated micro-LCD can be reduced, and a possible strategy is discussed in Fig. S18(B). In this step, we propose a waveguide-based laser back-illumination plate. An array of meta scatterers is patterned on one side, and grating couplers are patterned on the other side. The red, green, and blue laser light is first coupled into the waveguide through individual grating coupler. The coupled light is then guided through the plate and interacts with meta scatterers multiple times. The meta structures will scatter the guided wave into free space, and the light diffusion angle can also be engineered by meta scatterers. The scattered RGB laser light then illuminates onto the display after passing through a light diffuser. In this proposed setup, this laser illumination plate is more compact and has more engineering freedom to realize uniform back-light illumination.

References

1. Boyd, S., Boyd, S. P. & Vandenberghe, L. *Convex optimization*. (Cambridge university press, 2004).
2. Pestourie, R. *et al.* Inverse design of large-area metasurfaces. *Optics express* **26**, 33732-33747 (2018).
3. Svanberg, K. A class of globally convergent optimization methods based on conservative convex separable approximations. *SIAM journal on optimization* **12**, 555-573 (2002).

4. Kan, A. R. & Timmer, G. T. Stochastic global optimization methods part I: Clustering methods. *Mathematical programming* **39**, 27-56 (1987).
5. Harrington, R. F. *Time-harmonic electromagnetic fields*. (McGraw-Hill, 1961).
6. Bruno, O. P. & Pérez-Arancibia, C. Windowed Green function method for the Helmholtz equation in the presence of multiply layered media. *Proceedings of the Royal Society A: Mathematical, Physical and Engineering Sciences* **473**, 20170161 (2017).
7. Christiansen, R. E. *et al.* Fullwave Maxwell inverse design of axisymmetric, tunable, and multi-scale multi-wavelength metalenses. *Optics Express* **28**, 33854-33868 (2020).
8. Wirtinger, W. Zur formalen theorie der funktionen von mehr komplexen veränderlichen. *Mathematische Annalen* **97**, 357-375 (1927).
9. Hugonin, J. P. & Lalanne, P. Reticolo software for grating analysis. *arXiv preprint arXiv:2101.00901* (2021).
10. Lin, Z. & Johnson, S. G. Overlapping domains for topology optimization of large-area metasurfaces. *Optics express* **27**, 32445-32453 (2019).
11. Skarda, J. *et al.* Simulation of large-area metasurfaces with a distributed transition matrix method. *arXiv preprint arXiv:2107.09879* (2021).
12. Pérez-Arancibia, C., Pestourie, R. & Johnson, S. G. Sideways adiabaticity: beyond ray optics for slowly varying metasurfaces. *Optics express* **26**, 30202-30230 (2018).
13. Chung, H. & Miller, O. D. High-NA achromatic metalenses by inverse design. *Optics express* **28**, 6945-6965 (2020).
14. Chen, W. T. *et al.* Broadband achromatic metasurface-refractive optics. *Nano letters* **18**, 7801-7808 (2018).
